# Microglia-specificity of different markers is overridden in glioblastoma specimens

**DOI:** 10.1038/s41598-026-52315-y

**Published:** 2026-05-09

**Authors:** Alexander D. Bungert, Aminaa Sanchin, Anne Blank, Monika Jüngst-Mieczkowska, Annett Müller, Matthäus Felsenstein, Kati Turkowski, Wenying Zhang, Peter Vajkoczy, Susan Brandenburg

**Affiliations:** 1 Department of Experimental Neurosurgery, Charité – Universitätsmedizin Berlin, Corporate Member of Freie Universität Berlin and Humboldt-Universität zu Berlin, Charitéplatz 1, 10117 Berlin, Germany; 2 Present Address: Koch Institute for Integrative Cancer Research, Massachusetts Institute of Technology, Cambridge, MA USA; 3 Department of General, Visceral and Transplant Surgery, University Hospital Muenster, Albert-Schweitzer-Campus 1 - W1, 48149 Muenster, Germany; 4 Department of Neurosurgery, Charité – Universitätsmedizin Berlin, Corporate Member of Freie Universität Berlin and Humboldt-Universität zu Berlin, Charitéplatz 1, 10117 Berlin, Germany

**Keywords:** Microglia, Macrophages, Glioblastoma, SALL1, TMEM119, P2RY12, Cancer, Neuroscience

## Abstract

Microglia are the resident immune cells of the central nervous system. When glioblastoma develops, microglia and peripheral macrophages accumulate within the tumor tissue. Since both cell populations presumably exhibit different functional properties with anti- and pro-tumor characteristics, it is essential to distinguish between these cell populations accurately. The widespread and extensive use of single-cell technologies allowed the discovery of novel microglial markers like *Sall1*, *Tmem119*, *P2ry12* and *Hexb* that were used for discrimination of populations. In our study, these markers were tested on protein level using immunofluorescence staining. This method permits easy identification by co-staining with IBA1 as lineage marker for myeloid cells. Bone marrow chimeras served as differentiation control. Staining showed ubiquitous marker expression of microglia in both murine naïve brains and human epilepsy specimens. However, experiments using a murine glioblastoma model demonstrated that macrophages can also express these markers after infiltrating the brain and tumor tissue. Furthermore, the level of expression varies spatially, decreasing towards the intratumoral area. In vitro experiments confirmed positive staining results for both microglia and macrophages. Furthermore, cultivation with tumor-conditioned medium led to downregulation of microglial markers. Consequently, the concept that SALL1, TMEM119, P2RY12, and HEXB are exclusive markers for microglia depends strongly on the experimental and pathological conditions, and should not be generalized. Under neuroinflammatory conditions, cell specificity of the examined markers largely diminishes and both microglia and macrophages show ubiquitous expression. Considering this fact should lead to a context dependent application when using these markers to identify myeloid cell populations within the brain.

**Supplementary Information:**

The online version contains supplementary material available at 10.1038/s41598-026-52315-y.

## Introduction

Microglia are the resident immune cells of the central nervous system (CNS). They originate from progenitor cells of the yolk sac, and are necessary to maintain brain homeostasis and to respond to pathological stimuli^1^. Macrophages are bone-marrow derived and migrate to the CNS induced by brain pathology or damage^2^. Especially in glioblastoma, there is an extensive accumulation of both microglia and macrophages and these cells make up to 50% of the total tumor mass^3^. These infiltrates are often summarized as tumor-associated microglia/macrophages (TAMs). TAMs are recruited to the tumor by cytokines exerting anti- as well as pro-tumoral properties with spatial and temporal distinctions^4–10^. It can be assumed that precise differentiation of the cell populations is essential for targeted therapy due to different and contradictory functions in the tumor context. Diverse localization and infiltration pattern of both cell types also suggests nonredundant functions^11–13^.

To discriminate between microglia and macrophages is difficult due to sharing of the same myeloid lineage markers^14,15^. However, CD45 expression level was frequently used for differentiation between CD45^low^ microglia and CD45^high^ macrophages. But this dichotomy turned out to be too imprecise as there are studies that also prove CD45^high^ microglia under specific conditions^16–20^. Further methods of distinction are bone marrow chimeras, generated by total body (TBI) or head protected (HPI) irradiation and following transplantation of fluorescence labelled bone marrow cells. After a certain time span of reconstitution, circulating monocytes/macrophages are distinct from resident brain microglia via fluorescence signal^17,19,21,22^.

Another chimeric model is the parabiosis model. Therefore, two different organisms are connected temporarily creating a joint circulatory system providing a fluorescence signal as discrimination marker. Chimerism rate itself is usually relatively low and consequently underestimates the myeloid cell engraftment to the brain^23^.

All in all, these methods are highly demanding, require a long lead time and are cost-intensive. Recently, methods like single-cell technologies revealed new protein markers for macrophages such as CCR2, CD45RA, CD141, ICAM, CD207 and CD209^2,24–26^ as well as for microglia such as SALL1, TMEM119, P2RY12 and HEXB^27–32^. They promise easy and time-saving application. Here, we want to focus on the previously mentioned microglia markers.

SALL1 is a zinc-finger transcription factor expressed during embryogenesis of the CNS, heart, limbs or kidneys. It is critical for proper organogenesis. As a microglia specific signature gene, it was first described by Buttgereit et al.^27^. The authors showed specific protein expression in the murine CNS under physiological conditions in Sall1 reporter mice (Sall1^GFP^/^+^ mice) corresponding CD45^low^ microglial cells in flow cytometry. This expression behavior was supposed to be stable under neuroinflammatory circumstances using the experimental autoimmune encephalomyelitis model (EAE). Here, congenic bone marrow chimeras using Sall1^GFP/+^ CD45.1➙ CD.45.2 WT and WT CD45.1➙ Sall1^GFP/+^ CD45.2 mice could clearly discriminate peripherally recruited macrophages from central microglia via CD45.1/C45.2 marker system in flow cytometry. GFP fluorescence indicating SALL1 protein expression was only measured in microglial cells^27^.

TMEM119, also known as osteoblast induction factor, is a member of the transmembrane family TMEM that has been found to be upregulated in various cancers, such as prostate cancer, gastric cancer, breast cancer or osteosarcoma and correlates with a poor survival^33^. However, its function in microglia is still widely unknown^34^. It was found to be microglia specific in humans by analysis of five comprehensive datasets of mouse transcriptome^35–40^ by Satoh et al.^28^. These datasets were mostly generated by samples of purified CD45^low^ microglia in the healthy brain, either compared to the whole CNS, other CNS cell types or other myeloid cell compartments like spleen, lung or peritoneal cavity. Then, TMEM119 positivity of microglia was shown in human neurodegenerative diseases whereas infiltrating myeloid cells were considered as macrophages solely based on morphological and topographical characteristics identified by immunohistochemistry^28^.

P2RY12 serves as a metabotropic purinergic receptor of the microglial cell surface. It responds to adenosine triphosphate (ATP) released or secreted by injured cells^41^ and is present on microglia or astrocytes in both human and rodents^42,43^. P2RY12 is important for communication between microglia and neurons by regulating microglial chemotaxis in response to ATP^44^. P2RY12 is mainly membrane bound but shifts to the core in high-grade gliomas. Furthermore, cytoplasmatic expression is associated with the expression of M1 markers whereas nuclear P2RY12 correlates with protumoral M2-like expression patterns^45^. It was, amongst others, identified by Haage et al. as a signature gene for murine microglia^31,41^. This marker was further tested by proteomic analysis comparing central microglia to peripheral spleen monocytes/macrophages confirming P2RY12 as a valid microglia marker since expression in peripheral macrophages is hardly detectable. Additionally, different murine tumor models were applied. In the RCAS model, tumor-associated microglia were defined as usual by CD45^low^CD11b^+^ expression and monocytes/macrophages were determined by CD45^high^CD11b^+^. RNA sequencing revealed that P2RY12 was identified as being highly expressed solely in the CD45^low^ population assumed as microglia. Further experiments used mice with a hematopoietic lineage trace system^46^. Here for the RCAS and GL261 model, the gene expression showed significant enrichment of P2RY12 in the defined microglia population compared to peripheral monocytes/macrophages^31^.

Hexosaminidase B (HEXB) is the beta subunit of the lysosomal enzyme β-hexosaminidase degrading various cellular substrates. Loss of function leads to neurodegenerative diseases^47^. In literature, *HexB* was stated as microglial core gene crystallized by massive transcriptomic analysis^32^. It was shown that gene expression was stable in four different disease models (neurodegenerative and neuroinflammatory) without using tumor models. HEXB expression was observed on protein level by creating Hexb^tdT^ mice which results in HEXB^+^ microglia expressing the tdTomato (tdT) fluorescent protein^32^. By using transgenic HEXB^tdT/tdT^CX3CR1^GFP/+^ mice microglia should be distinguishable from macrophages with microglia showing double positivity for tdT and GFP and macrophages for GFP only.

In this study, we tested the microglia markers SALL1, TMEM119, P2RY12 and HEXB on human and mouse specimens under naïve and glioma conditions. We show whether they are expressed on protein level and if they can be considered as specific markers to discriminate microglia from macrophages under tumor conditions. As gold standards for differentiation of microglia and macrophages, we used bone marrow chimeras generated by total-body irradiation (TBI) and head-protected irradiation (HPI). Moreover, we evaluated primary microglia and macrophages for marker expression *in vitro.* Additionally, we stimulated the microglial cell line BV2 with tumor-conditioned medium (TCM) to investigate their response concerning expression of SALL1, TMEM119, P2RY12 and HEXB.

## Materials and methods

### Human specimens

Brain tissue samples of five epilepsy patients were collected during therapeutic surgery in 2014 (Department of Neurosurgery, Charité-Universitätsmedizin Berlin, Germany). Patient characteristics: gender – 3/2 (f/m); age – 41.6 ± 15.3 years. All human experiments were conducted in accordance with relevant guidelines and regulations (including institutional, national, and international ethical standards). The study received formal ethical approval by the Ethical Committee of Charité-Universitätsmedizin Berlin (application number: EA4/065/13). Written informed consent was obtained from all participants prior to sample collection and analysis.

### Animal work

#### Mice

Mice were housed in a 12/12-hour light-dark cycle with food and water provided ad libitum. Male mice (weight: 20–38 g; age: 8–52 weeks) were used for experiments. All experiments were performed according to the German Law for Animal Protection and the National Institute of Health Guidelines for Care and Use of Laboratory Animals. For animal experiments, we followed the ARRIVE guidelines. Animal studies were approved by the local ethics committee on animal research, LaGeSo Berlin (Permit Number: T0159/09, G152/09, G0376/11 and G0281/14). C57BL6/J mice (gender: male, age: 10–18 weeks, weight: 25–34 g, wildtype and as recipients for bone marrow; gender: male, age: 8–28 weeks, weight: 20–35 g as donor for bone marrow) were purchased from Charles River (Sulzfeld, Germany). Heterozygous C57BL6/J-TgN(beta-act-EGFP) mice (gender: male, age: 8–52 weeks, weight: 20–38 g as donor for bone marrow) and heterozygous Cx3cr1^+/−^-GFP (B6.129P2(Cg)-Cx3cr1tm1Litt/J) mice (gender: male, age: 10–18 weeks, weight: 25–34 g as recipient for bone marrow; gender: male, age: 10–48 weeks, weight: 22–38 g as donor for bone marrow) were purchased from Charles River and bred in-house (FEM, Berlin, Germany). Genotyping was performed for every transgenic animal. The recipient animals were matched according to sex, age and weight.

Animals were randomly assigned to groups and analyses were conducted blinded. The animal experiments were repeated two to three times to achieve a total of 5–6 animals per group.

#### Generation of bone marrow chimeras

Total body irradiated (TBI) chimeras were generated by standard procedure^48,49^. Mice (C57BL6/J) were anesthetized (90 mg/kg ketamine, Ketavet, Pfizer; 10 mg/kg xylazine, Rompun, Bayer HealthCare; intraperitoneal injection) and lethally irradiated (11.5 Gy) with cesium 137 (Gammacell^®^ 40 Exactor from MDS Nordian, Deutsches Rheuma Forschungszentrum, Berlin, Germany). For HPI, animals (C57BL6/J or Cx3cr1^+/−^-GFP) were placed into a lead shield construction followed by irradiation^17^. Within 12 h, freshly collected 1.7-2.0 × 10^7^ bone marrow cells of either C57BL6/J-TgN(beta-act-EGFP) or C57BL6/J were injected intravenously (200µL/animal). After 8 weeks, reconstitution levels were examined by flow cytometry of blood samples as described before^17,19^. Only animals with a reconstitution level of above 75% of CD45^+^CD11b^+^ cells were further analyzed (reconstitution level: 75–95%).

#### Stereotactic tumor cell implantation

Eight weeks after the generation of bone marrow chimeras, we injected GL261 tumor cells intracranially. Anaesthetized mice (90 mg/kg ketamine; 10 mg/kg xylazine; intraperitoneal injection) were fixed in a stereotactic frame (Stoelting). Bepanthen eye cream (5% Dexpanthenol, Bayer) was used to cover the eyes during surgery. To prevent pain, the scalp was treated with lidocaine (Xylocain pump spray, Aspen). A midline incision was done on the scalp, a hole was drilled by a canula and tumor cells were injected 1 mm anterior and 2 mm lateral to the bregma. Here, a microliter Hamilton syringe (1µL; Carl Roth GmbH, Karlsruhe, Germany) was inserted into 4 mm depth, returned to 3 mm, and cells were slowly released (2.0 × 10^4^ cells/µL). After surgery, Paracetamol (300 mg/kg, Paracetamol-ratiopharm solution, Ratiopharm) was added to the drinking water for pain management (up to 72 h). Animals were monitored at least twice daily for the first 72 h for postoperative signs of distress. The endpoint of the study was day 21 after tumor cell implantation.

#### Magnetic resonance imaging (MRI)

Magnetic resonance imaging was performed on day 21 after GL261 tumor cell inoculation. Tumor-bearing mice were anesthetized by 1.5–2.0% isoflurane (Forene, Abbot, Wiesbaden, Germany) delivered in a O_2_/N_2_O mixture (30%/70%) and placed into a 7-Tesla rodent MRI scanner (Pharmascan 70/16, Bruker BioSpin, Rheinstetten, Germany). Brains were observed with T2-weighted two-dimensional turbo spin-echo sequences by Paravision 4.0 software (Bruker BioSpin). Calculation of tumor volumes was carried out with the program Analyze 5.0 (AnalyzeDirect, Lenexa, KS). Tumor volumes were as follows. Wildtype animals (WT, *n* = 5): 9.83 ± 5.20 mm^3^; total body irradiated chimeras (TBI, *n* = 5): 21.52 ± 10.00 mm^3^; head protected irradiated animals (HPI, *n* = 6): 9.60 ± 4.75 mm^3^. As expected, the tumor volumes of WT and HPI animals were comparable, while the tumor sizes of TBI mice doubled^17^.

#### Isolation of bone marrow cells

Naïve animals were anesthetized with isoflurane (Isofluran CP, Cp-Pharma) and sacrificed by cervical dislocation. Bone marrow cells were harvested by flushing femurs of C57BL6/J or Cx3cr1^+/−^-GFP mice with Ca^2+^-, Mg^2+^-free phosphate-buffered saline (PBS; PAA Laboratories). Suspensions were centrifuged and passed through a pre-separation filter (30 μm; Miltenyi Biotec, Bergisch Gladbach, Germany) to obtain single-cell suspensions. Viable cells were diluted in PBS to a concentration of 1.7–2.0 × 10^7^ cells/200 µL.

#### Isolation of microglia from adult brains

Naïve mice were anaesthetized (90 mg/kg ketamine; 10 mg/kg xylazine; intraperitoneal injection) and perfused intracardially with PBS. Brains were taken and processed under sterile conditions. Brains were homogenized by using the Neural Tissue Dissociation Kit (P) (Miltenyi Biotec) and the gentleMACS™ Octo Dissociator with Heaters (Miltenyi Biotec). Isolation of microglia was performed by addition of CD11b MicroBeads (Miltenyi Biotec) and two subsequently used columns (LS column, MS column; Miltenyi Biotec). Purity of microglia was analyzed by FACS (BD FACS CantoII) following staining with CD11b-PE (Biolegend) and CD45-APC (BD Biosciences) antibodies. Purity of eluted cells was approx. 95% CD45^+^CD11b^+^ cells.

### Cell culture

#### Cultivation of GL261 cells for implantation

GL261 cells (passage 14) were grown in high-glucose Dulbecco’s modified Eagle’s medium (DMEM) containing stable glutamine and sodium pyruvate (PAA Laboratories) supplemented with 10% fetal calf serum (PAA Laboratories), 100 units of penicillin/ml and 100 µg/mL of streptomycin at 37 °C and 5% CO_2_ atmosphere. The GL261 cell line, an established murine glioblastoma model, was obtained from the German Collection of Microorganisms and Cell Cultures (DSMZ, ACC802), Germany. Cells were cultured for three days until a confluence of 80% was reached. Cells were detached using 0.5% trypsin/EDTA (Life Technologies, Darmstadt, Germany), washed with PBS and diluted to a concentration of 2 × 10^4^ cells in 1 µL.

#### Preparation of tumor-conditioned medium

GL261 cells were cultured as described above but with 5% FCS. After three days in culture (confluence approx. 80%), supernatant was collected and centrifuged two times at 4000 rpm to exclude cells and debris.

#### Differentiation and cultivation of macrophages

Bone marrow cells were seeded in a T75 flask (Sarstedt) using RPMI 1640 medium (Gibco, ThermoFisher Scientific) containing stable glutamine with 10% fetal calf serum, 100 units of penicillin/ml and 100 µg/ml of streptomycin. Additionally, M-CSF (20 ng/mL; Miltenyi Biotec) was added and medium change was performed every second or third day for eight days. Macrophages attached to the bottom. On day eight of culture, macrophages were detached by a cell scraper, washed and plated in 8-well chamber slides (x-well cell culture chamber, 8 wells, on PCA slide; Sarstedt). 1.2 × 10^5^ cells/well were plated in RPMI medium including supplements for two days.

#### Plating of primary microglia

Primary isolated microglia were seeded into 8-well chamber slides (x-well cell culture chamber, 8 wells, on PCA slide). Cells were cultured for three days in high-glucose Dulbecco’s modified Eagle’s medium (DMEM) containing stable glutamine and sodium pyruvate supplemented with 10% fetal calf serum, 100 units of penicillin/ml and 100 µg/ml of streptomycin at 37 °C and 5% CO_2_ atmosphere. 2.5 × 10^5^ cells/well were plated in DMEM medium including supplements for three days.

#### Cultivation and plating of BV2 cells

BV2 cells (passages 5–6), an established immortalized murine microglial cell line, were used. The cell line was kindly provided by PD Dr. Marina Jendrach (Department of Neuropathology, Charité – Universitätsmedizin Berlin, Germany). Cells were cultured in high-glucose Dulbecco’s modified Eagle’s medium (DMEM) containing stable glutamine and sodium pyruvate supplemented with 5% fetal calf serum, 100 units of penicillin/ml and 100 µg/ml of streptomycin at 37 °C and 5% CO_2_ atmosphere. Cells were grown for three days. Cells were detached using 0.5% trypsin/EDTA. Cells were counted and 7,500 cells were plated for each well of an 8 well-chamber slide (x-well cell culture chamber, 8 wells, on lumox^®^ slide, Sarstedt). DMEM with 5% FCS or tumor-conditioned medium (TCM) was used. After three days of culture, cells were fixed and stained.

### Immunofluorescence staining

#### Processing, immunofluorescence staining and analyses of human tissue samples

Directly after surgery, human brain tissues were fixed in 4% PFA for 24 h and dehydrated in rising sucrose concentrations (10%/20%/30%; Roth, Karlsruhe, Germany). The brain tissue samples were carefully frozen with liquid nitrogen. Frozen sections were prepared at 10 μm thickness.

All sections were treated with Autofluorescence Eliminator Reagent (Millipore, Burlington, MA, USA) following the instructions of the manufacturer. Afterwards, human brain sections were permeabilized by 0.1% Triton X-100/PBS (20 min, room temperature; SALL1, P2RY12, HEXB) or directly blocked with 1% Casein/PBS (30 min, RT; TMEM119). Sections were stained with primary antibodies for 2 h at RT. To identify microglia/macrophages, IBA1 staining (goat anti-IBA1, Abcam, Cambridge, UK, ab5076, 1:200) was performed. Additionally, the following antibodies to detect specific markers were used: rabbit anti-SALL1 (1:100; PA5-62057, ThermoFisher Scientific), rabbit anti-TMEM119 (1:100; ab185337, Abcam), rabbit anti-P2RY12 (1:100; NBP2-33870, Novus Biologicals), rabbit anti-HEXB (1:100; PA5-101082, ThermoFisher Scientific). After washing section  3 × 5 min in 0.5% Casein/PBS, secondary antibodies were applied (AF647-conjugated anti-goat IgG; Cy3-conjugated anti-rabbit IgG; Dianova, Hamburg, Germany, each 1:200). After incubation for 1.5 h at RT, sections were washed 2 × 5 min in PBS and water. DAPI-containing mounting medium (Immunoselect Antifading Mounting Medium DAPI, Dianova) was used to stain nuclei.

Images were acquired using a Zeiss Axio Observer Z1 fluorescence microscope (Zeiss MicroImaging GmbH, Jena, Germany) at room temperature. Approximately 24 images for each patient at three different brain tissue areas were taken. ImageJ 1.54j (NIH, Bethesda, MD, USA) was used to analyze images.

#### Processing of murine brains, and immunofluorescence staining and analyses of sections

For immunohistochemically studies, on day 21 of tumor growth, glioma-bearing mice were anaesthetized (90 mg/kg ketamine; 10 mg/kg xylazine; intraperitoneal injection) and perfused intracardially with 4% paraformaldehyde (PFA). Brains were fixed in 4% PFA and subsequently dehydrated by sucrose solutions in rising concentrations (10%/20%/30%). Frozen brains were embedded in gelatine and sliced by a cryostat (Microm HM 560, Microm International GmbH) in coronal sections of 10 μm thickness.

For staining of TMEM119, sections were directly blocked with 1% Casein/PBS (30 min, RT). For staining of SALL1, sections were fixed with methanol (10 min, -20 °C) and blocked, subsequently. For P2RY12 and HEXB staining, sections were treated with Antigen Retrieval Reagent-Universal (VCTS023, R&D Systems; heating up to 90 °C, 2–5 min) and blocking was performed with 0.1% Triton X-100 in 1% Casein/PBS (30 min, RT). Primary antibodies were used to detect microglia/macrophages (IBA1), and the different markers: goat anti-IBA1 (1:200; ab5076, Abcam), rabbit anti-SALL1 (1:100; ab31526, Abcam), rabbit anti-TMEM119 (1:50; ab209064, Abcam), rat anti-P2RY12 (1:100; 848002, Biolegend), rabbit anti-HEXB (1:100; PA5-101082, ThermoFisher Scientific). Following 2 h of incubation at room temperature, sections were washed with 0.5% Casein (4 × 5 min). Secondary antibodies were applied for 1.5 h: AF647-conjugated anti-goat IgG; Cy3-conjugated anti-rabbit IgG, Cy3-conjugated anti-rat IgG (1:200; Dianova). Sections were washed 3 × 5 min in PBS and water. DAPI-containing mounting medium (Dianova) stained nuclei.

Images were acquired using Zeiss Axio Observer Z1 fluorescence microscope at RT. Up to 15 images for each brain region of three different sections were taken (animals: 4–6 in total per group). Images were analyzed by ImageJ 1.54j (NIH).

Negative controls were performed using secondary antibodies alone. For each condition, two to three brain sections from two different TBI mice were included and processed according to the same staining protocols as the experimental samples. Imaging (three regions per section: contralateral, peritumoral, and intratumoral), as well as image processing and analysis, were performed using the same settings and workflow as for the corresponding immunostainings.

#### Fixation and staining of cultured cells (primary microglia, macrophages and BV2 cells)

Primary microglia, macrophages and BV2 cells were fixed with 4% PFA (RT, 20 min). For SALL1 and TMEM119 blocking was performed with 1% Casein/PBS. For P2RY12 and HEXB blocking was performed with 0.1% Triton X-100 (SigmaAldrich) in 1% Casein/PBS (RT, 30 min) The following primary antibodies were used for staining: rabbit anti-SALL1 (1:100; Abcam), rabbit anti-TMEM119 (1:50; Abcam), rat anti-P2RY12 (1:100; Biolegend), rabbit anti-HEXB (1:100; ThermoFisher Scientific), goat anti-IBA1 (1:100; Abcam) or Phalloidin (1:200; Alexa Fluor™ 488 Phalloidin, A12379, Invitrogen). Incubation of primary antibodies was 2 h at RT. Following washing, secondary antibodies were applied (dilution for each antibody was 1:200): Cy3-conjugated anti-rabbit IgG, Cy3-conjugated anti-rat IgG, AF647-conjugated anti-goat IgG (Dianova). DAPI-containing mounting medium was used to stain nuclei.

Images were acquired using Zeiss Axio Observer Z1 fluorescence microscope at RT. Approx. 8 images were taken for each well (4–11 wells/marker). Images were analyzed by ImageJ 1.54j (NIH).

Negative controls for primary microglia/macrophages and BV2 cells were performed using secondary antibodies alone. For each condition, one to two wells were included, and 5–8 images were acquired per well. Imaging and subsequent image processing were performed using the same settings and workflow as for the corresponding stained samples.

### Statistics

Statistical analyses were performed using GraphPad Prism (GraphPad Software 10). Depending on the experimental setup, comparisons between two groups were conducted using unpaired Student’s t-test, whereas comparisons among multiple groups were analyzed using one-way analysis of variance (ANOVA) followed by Bonferroni post hoc correction. Data are presented as mean ± standard deviation. A p-value of < 0.05 was considered statistically significant.

## Results

### Naïve human and murine microglia show high co-expression of SALL1, TMEM119 and P2RY12

**Fig. 1:**
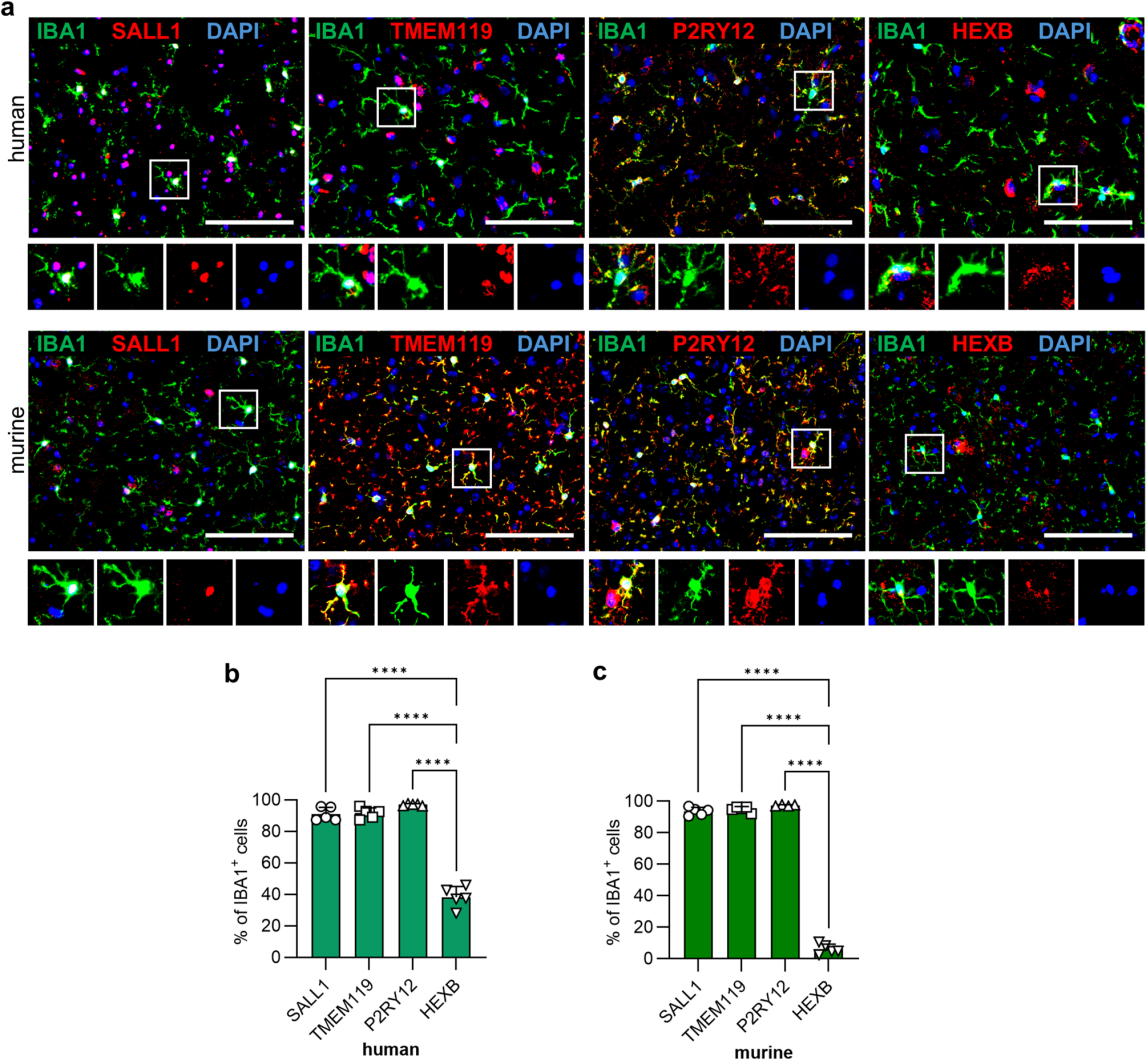
Naïve microglia show high coexpression with various markers in both human and murine brains. **a** Sections of human brain samples from epilepsy patients (upper row) and sections of naïve murine brains (lower row) were stained for IBA1 (green; microglia/macrophages) and the respective markers (red). DAPI (blue; nuclei). Representative images of each staining are depicted. *Scale bars* 100 μm. *Squares* indicate magnified cells. **b** Calculation of marker positive IBA1^+^ cells of human brain specimens is shown (*n* = 5). **c** Graph depicts expression of markers of IBA1^+^ cells within the naïve murine brain (*n* = 4–5). ANOVA and Bonferroni correction, p**** < 0.0001.

We analyzed the four markers SALL1, TMEM119, P2RY12 and HEXB, described as specific for microglial cells, by immunofluorescence staining. Samples of human brains from epilepsy (EP) patients and tissues of naïve murine brains were used to define their co-expression with IBA1^+^ microglia. In both species, we found microglia that express the respective markers (Fig. 1a). All markers examined in the sections exhibited the expected pattern of expression. In human specimens, co-expression with IBA1 is very high showing an average of 91.0% for SALL1, 91.6% for TMEM119, 97.0% for P2RY12 and the lowest for HEXB at 38.2% (Fig. 1b). In naïve mouse brains a similar distribution of marker expression can be detected. SALL1 showed 93.6%, TMEM119 95.0% and P2RY12 97.4%. Here, HEXB is also the marker with the lowest co-expression with IBA1^+^ cells of only 6.1% (Fig. 1c). Thus, as expected from previous gene expression analyses, SALL1, TMEM119 and P2RY12 seem to be good markers for microglia under almost homeostatic conditions on protein level in human and murine brain tissues. In contrast, HEXB defined the microglia population inadequately in both species. Furthermore, it should be noted that, in addition to IBA1-positive cells, other cells also showed positive staining in the analyzed sections, indicating limited specificity of these markers.

### Microglia marker expression in murine glioblastoma is determined by its intracranial localization

**Fig. 2:**
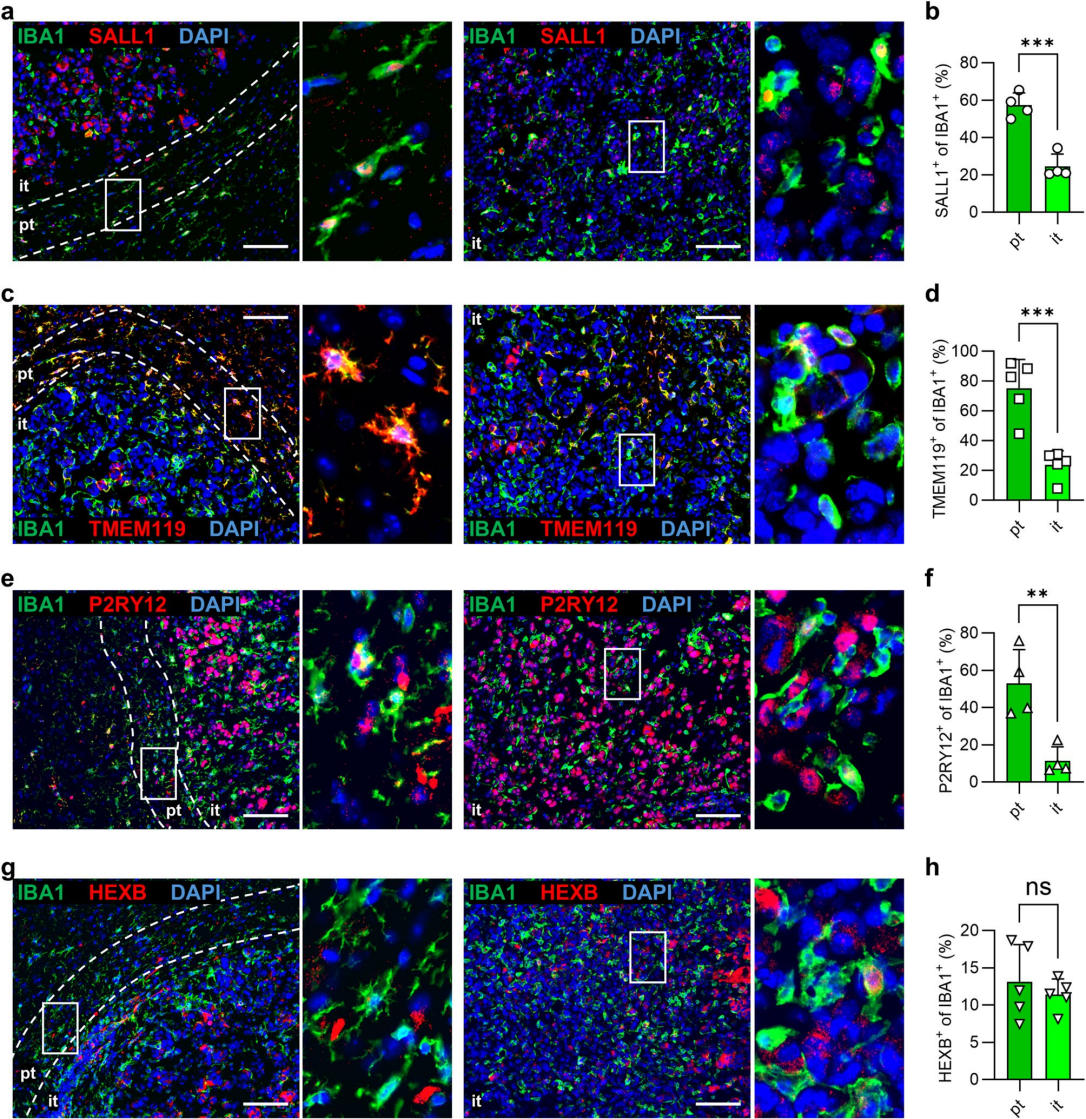
Expression of SALL1, TMEM119 and P2RY12 by IBA1^+^ cells is reduced in the intratumoral area of murine glioblastoma. GL261 murine glioblastoma cells were used for intracranial inoculation. Brains were harvested at day 21 of tumor growth. **a**,** c**,** e**,** g** Murine sections of glioblastoma tissues were stained for IBA1 (green; microglia/macrophages), DAPI (blue; nuclei) and the respective marker (red): SALL1 (**a**), TMEM119 (**c**), P2RY12 (**e**), HEXB (**g**). **b**,** d**,** f**,** h** Graphs show calculation of SALL1^+^ cells (**b**), TMEM119^+^ cells (**d**), P2RY12^+^ cells (**f**) and HEXB^+^ cells (**h**) within the IBA1^+^ population in different brain areas. *pt* peritumoral (100 μm around the tumor tissue; dashed line), *it* intratumoral. *Scale bars* 100 μm. *Rectangles* indicate magnified tissue areas. Student`s t-test was used (*n* = 4–5), p** < 0.01, p*** < 0.001, *ns* not significant.

Since there is high similarity between human and mouse brains in marker expression, we further analyzed the respective markers in brain tumors of mice. Glioblastoma cells were implanted intracranially and tumors were investigated on day 21 of glioma growth. We evaluated the intratumoral area (*it*) and the peritumoral area (*pt*), which contains the region of 100 μm around the tumor border. All markers were found within the peritumoral and intratumoral area (Fig. 2a, c, e, g). Interestingly, SALL1 (Fig. 2b), TMEM119 (Fig. 2d) and P2RY12 (Fig. 2f) showed significant reduction of expression from the peritumoral towards intratumoral area resulting in very low percentages intratumorally (SALL1 24.5%, TMEM119 23.8%, P2RY12 11.5%), while HEXB (Fig. 2h) depicted low percentage of IBA1^+^ cells in both locations. Thus, expression of SALL1, TMEM119 and P2RY12 is strongly reduced in the tumor tissue compared to the peritumoral area and contralateral hemisphere. Under these pathological conditions, additional positive staining was observed that did not correspond to IBA1-positive cells, especially for P2RY12.

### Markers are expressed by microglia but also by infiltrating macrophages in murine glioblastoma

**Fig. 3:**
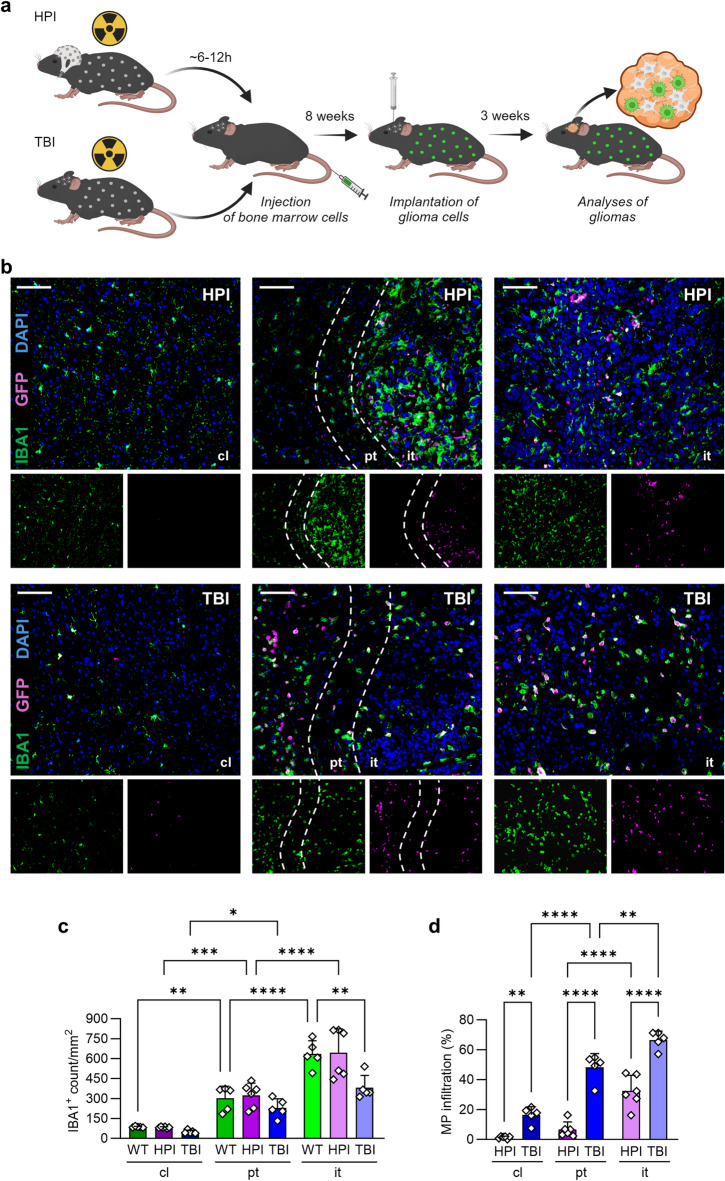
Two different chimeric models allow the discrimination of microglia and macrophages. **a** Scheme of chimera generation (created with BioRender.com). Bone marrow cells of recipients were depleted by head protected irradiation (HPI; upper row) or total body irradiation (TBI; lower row). Animals were reconstituted with bone marrow cells (GFP^+^ BM). Eight weeks after BM transfer GL261 tumor cells were implanted. 21 days following the implantation, brains were harvested. **b** Sections were stained for IBA1 (green; microglia/macrophages) and DAPI (blue; nuclei). GFP (violet) signal visualized brain infiltrating cells. Different regions of the murine brains are depicted (cl, pt, it). *Scale bars* 100 μm. *Dashed lines* define peritumoral area. **c** Graph depicts number of IBA1^+^ cells in different brain regions of animals without irradiation (WT), HPI and TBI treatment. **d** Graph represents infiltration of macrophages (MP; as part of total IBA1^+^ cells) in the HPI and TBI model. *WT* wildtype, *HPI* head protected irradiation, *TBI* total body irradiation, *MP* macrophages, *cl* contralateral, *pt* peritumoral, *it* intratumoral. ANOVA and Bonferroni correction (*n* = 5–6), p* < 0.05, p** < 0.01, p*** < 0.001, p**** < 0.0001.

The mentioned markers are recently used for discrimination of microglia and macrophages using RNASeq data^27–32^. Both cell populations accumulate in the tumor area^3^. However, the ratio of microglia and macrophages in glioma is still under discussion^18,50^. To evaluate if markers are co-expressed by solely microglia in the tumor area on protein level, we generated bone marrow chimeras. These chimeras allow the adequate discrimination of brain microglia (IBA1^+^GFP^−^; MG) and macrophages derived from bone marrow (IBA1^+^GFP^+^; MP) by GFP labelling. Here, we used two different models, the total body irradiation (TBI) and the head protected irradiation (HPI). Following irradiation, transfer of bone marrow cells, and a reconstitution time of eight weeks, tumor cells were implanted intracranially and glioblastoma tissue was analyzed on day 21 of growth (Fig. 3a). Both irradiation strategies revealed infiltration of GFP^+^ macrophages into the brain. Here, the HPI strategy showed solely GFP signal in the intratumoral region while TBI led to GFP signals in all depicted areas (Fig. 3b). Compared to untreated animals (WT) the amount of IBA1^+^ cells in the contralateral brain hemisphere was unaffected by irradiation with both methods. As expected, in all groups IBA1^+^ count increased in peritumoral area and showed highest numbers in the tumor region. Notably, WT and HPI animals revealed similar IBA1^+^ counts in all areas while in TBI we found a reduced number of IBA1^+^ cells in the intratumoral area compared to the non-irradiated brains indicating less accumulation of myeloid cells after total body irradiation (Fig. 3c). Previously, flow cytometric data revealed that the infiltration of macrophages is higher in TBI animals^17^. This could be verified by our immunofluorescence analyses. The macrophage influx of TBI mice in all three brain regions was significantly higher than in HPI mice. In the contralateral and peritumoral region of HPI mice, macrophages were barely present with 1.4% and 6.6% of all IBA1^+^ cells. In contrast, there was a significant increase of macrophages of TBI mice between the contralateral, peritumoral and intratumoral area with 16.4%, 48.4% and 66.6%. In the HPI model, macrophage infiltration also rose significantly, but only between the peri- and intratumoral area with 6.6% and 32.3% (Fig. 3d). Total body irradiation allows the analysis of marker expression by infiltrating macrophages in contralateral and peritumoral tissue, whereas in the HPI model these cells are largely confined to the tumor core and can therefore only be reliably quantified in that region.

**Fig. 4:**
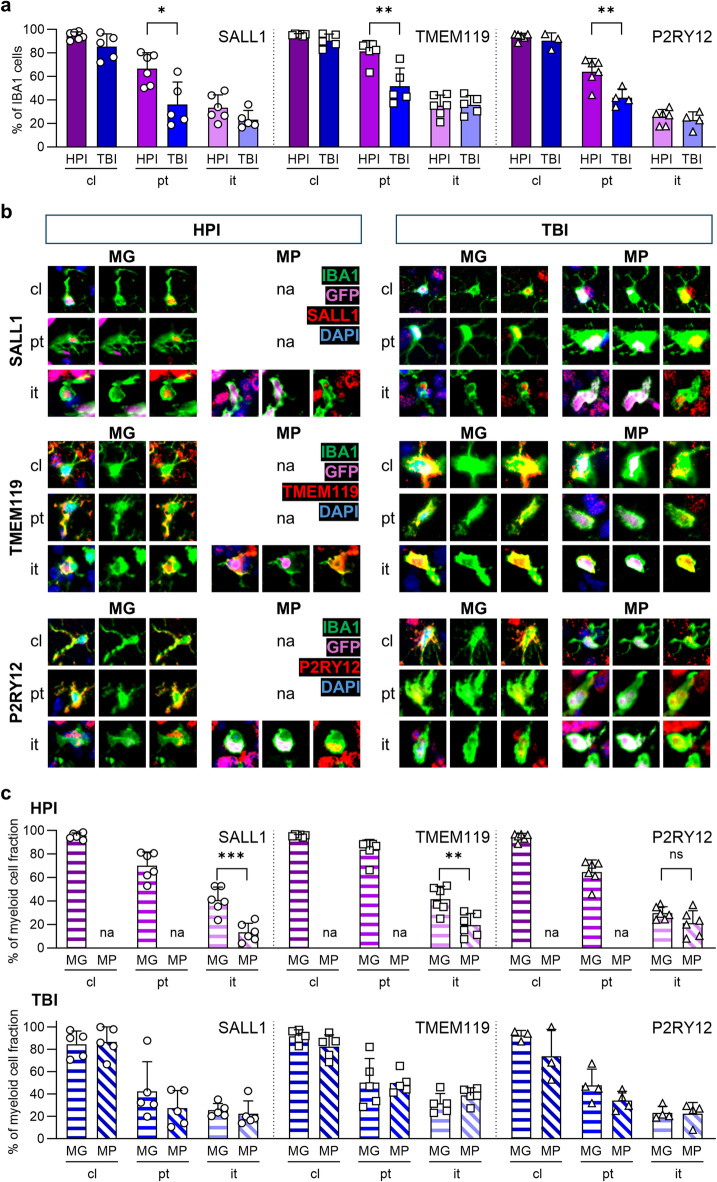
Microglia and macrophages in all brain regions express analyzed markers. Brain sections of HPI and TBI chimeras were stained for IBA1 (green; microglia/macrophages), and the respective markers (red). Microglia (MG) and macrophages (MP) were discriminated by GFP expression (MG: IBA1^+^GFP^−^, MP: IBA1^+^GFP^+^). **a** Graph depicts SALL1, TMEM119 and P2RY12 expression of all IBA1^+^ cells in different brain regions and under HPI and TBI condition. **b** Images show single cells of microglia and macrophages that express SALL1, TMEM119 and P2RY12. DAPI (blue, nuclei), GFP (violet, infiltrated cells). **c** Graphs depict calculation of microglia and macrophages that express SALL1, TMEM119 and P2RY12 in different brain regions under HPI (upper graph) and TBI (lower graph) condition. *HPI* head protected irradiation, *TBI* total body irradiation, *MG* microglia, *MP* macrophages, *cl* contralateral, *pt* peritumoral, *it* intratumoral, *na* not applicable. ANOVA and Bonferroni correction (*n* = 3–6), p* < 0.05, p** < 0.01, p*** < 0.001, *ns* not significant. An overview of the images and the corresponding negative controls is provided in Figs. S1 and S2.

We investigated HPI and TBIirradiated animals for marker expression, focusing on SALL1, TMEM119, and P2RY12, which showed high co-expression with microglia in naïve animals and therefore demonstrated potential as microglia-specific markers. In contrast, HEXB was not further pursued as a candidate marker due to its already limited colocalization with IBA1-positive microglia under naïve conditions. We observed for HPI and TBI animals, that expression of markers of IBA1^+^ cells is high in the contralateral hemisphere, showed reduction in the peritumoral area and achieved lowest level in the tumor tissue (Fig. 4a) as detected for brains without irradiation (Figs. 1c and 2). In total, the effect of irradiation is marginal on overall marker expression of IBA1^+^ cells. However, we found slight differences of marker expression in the peritumoral area. Here, the expression of SALL1, TMEM119 and P2RY12 was lower in TBI treated animals compared to HPI irradiation. Discrimination of microglia and macrophages by GFP signal revealed expression of SALL1, TMEM119 and P2RY12 by both myeloid cell populations with a similar expression pattern independently of the irradiation strategy (Fig. 4b). Remarkably, based on the low numbers of infiltrated macrophages within the contralateral hemisphere and peritumoral area following HPI treatment, their co-expression with markers could not be analyzed. Intratumorally, we observed that only 23–41% of the microglia population expressed SALL1, TMEM119 and P2RY12 following HPI and TBI irradiation (Fig. 4c). Importantly, macrophages showed also expression of these three markers to high extent within the contralateral hemisphere and peritumoral area following TBI. In the tumor region 13–39% of macrophages expressed SALL1, TMEM119 and P2RY12. Principally, the expression of markers between microglia and macrophages within the different brain areas was comparable. Solely intratumorally, we detected a significant difference between microglia and macrophages of SALL1 and TMEM119 expression following HPI treatment. Here, microglia expressed the markers to a higher extent than macrophages.

### Microglia markers are expressed by primary microglia and macrophages in vitro and downregulated in BV2 microglial cells under tumor conditions

**Fig. 5:**
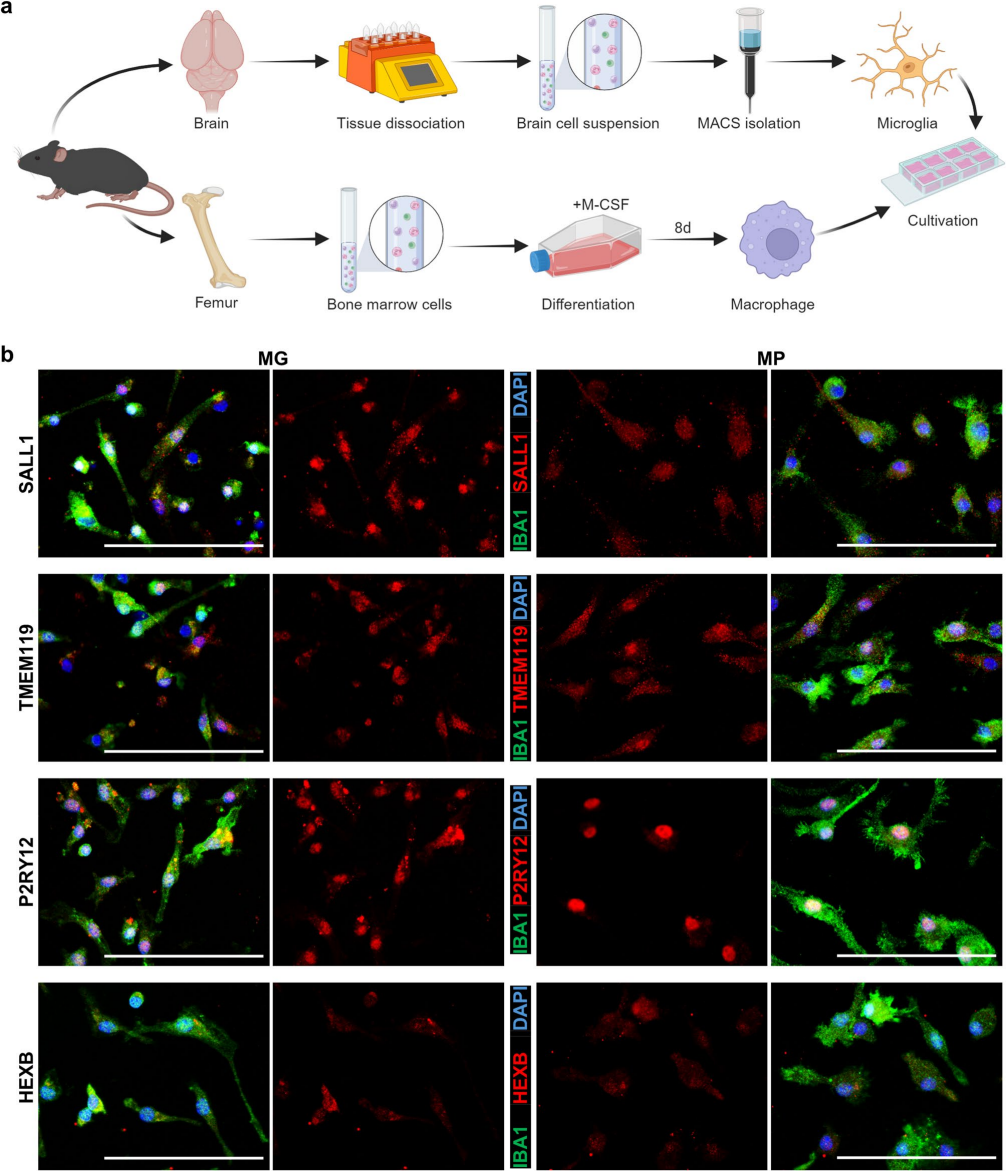
Primary microglia and primary macrophages express markers in vitro. **a** Scheme of the isolation and culture procedure for primary microglia and bone marrow-derived macrophages (created with BioRender.com). Microglia were isolated from naïve mouse brains and cultured for three days followed by fixation and staining. In addition, bone marrow cells were isolated, and cultured for eight days with M-CSF for differentiation of macrophages. Macrophages were taken and plated. After two days of culture, macrophages were fixed and stained. **b** Microglia and macrophages were stained for IBA1 (green; microglia/macrophages) and the respective markers (red). DAPI (blue; nuclei). *Scale bars* 100 μm. *MG* microglia, *MP* macrophages. Representative pictures of two independent experiments are shown (3–4 wells/marker in total, 8 images/well). An overview of the images and the corresponding negative controls is provided in Fig. S3.

**Fig. 6:**
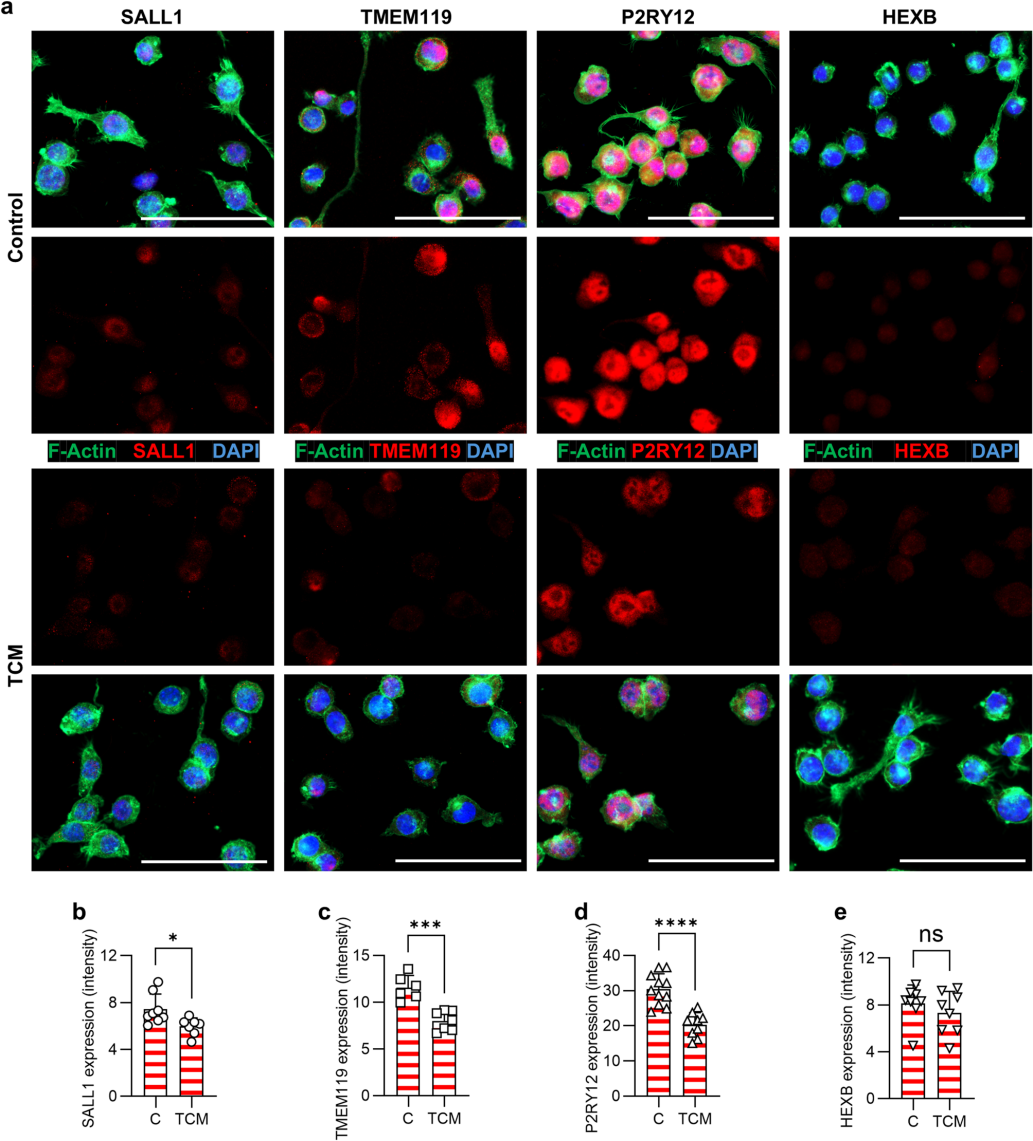
Microglial cells (BV2) downregulate markers after stimulation with tumor-conditioned medium. BV2 cells were cultured for three days in standard medium (Control) or tumor-conditioned medium (TCM). **a** Cells were fixed and stained with Phalloidin (green, F-Actin) and the respective markers (red). DAPI (blue, nuclei). *Scale bars* 100 μm. Representative images of three independent experiments are depicted. **b-e** Mean intensity of fluorescence was measured for each marker. Graphs depict intensity calculation for SALL1 (**b**), TMEM119 (**c**), P2RY12 (**d**) and HEXB (**e**). *C* control, *TCM* tumor-conditioned medium. Student`s t-test was used (*n* = 6–11 wells/marker in total), p* < 0.05, p*** < 0.001, p**** < 0.0001, *ns* not significant. An overview of the images and the corresponding negative controls is provided in Fig. S4.

To verify the expression of SALL1, TMEM119, P2RY12 and HEXB by microglia and macrophages, we investigated primary murine microglia and macrophages *in vitro.* We isolated microglia from naïve mouse brains by MACS technology and extracted bone marrow cells from mouse femurs and differentiated these cells by supply of M-CSF into macrophages (Fig. 5a). Staining revealed that all markers were expressed by both microglia and macrophages (Fig. 5b). Expression by macrophages was slightly lower than by microglia. HEXB showed the lowest and P2RY12 the highest expression of the markers in vitro. Furthermore, we cultivated BV2 microglial cells under non-stimulated conditions and with tumor-conditioned medium (TCM). This microglial cell line expressed all analyzed markers whereby P2RY12 depicted highest expression, and SALL1 and HEXB the lowest (Fig. 6a). A significant downregulation of expression was detectable for SALL1 (Fig. 6b), TMEM119 (Fig. 6c) and P2RY12 (Fig. 6d) of the TCM treated BV2 cells. HEXB expression was unaffected by cultivation with TCM (Fig. 6e). These in vitro data are consistent with the in vivo data, where markers are downregulated in the tumor area except for HEXB.

## Discussion

TAMs represent a large cell population within glioblastomas^51^. Therefore, there is a strong interest of analyzing and differentiating the exact composition of these cells, eventually creating druggable targets. Due to new techniques such as single-cell analysis, there are now a variety of markers that can be used to distinguish microglia from recruited macrophages based on their gene expression. However, expression analysis at the protein level in glioma context remains scarce. In this study, we examined the potential microglia markers SALL1, TMEM119, P2RY12 and HEXB via immunofluorescence staining on human and mouse specimens under naïve and glioma conditions. Moreover, we tested the expression behavior of primary microglia and macrophages in vitro under naïve conditions. Additionally, we examined the impact of TCM on BV2 microglial cells with regard to microglial specific marker expression. First, we can conclude that microglia in naïve murine and human brains express the examined markers to a high extent with more than 90%, except for HEXB with considerably lower co-expression. This indicates that microglia can be defined by SALL1, TMEM119 and P2RY12 in naïve brains by immunofluorescence staining. Conversely, within glioma tissue, we observed a greatly reduced number of myeloid cells that expressed the markers on protein level. This was expected due to additional infiltration of peripheral macrophages that were postulated as negative for the analyzed markers^27–32^. For the subsequent bone marrow chimeric experiments, we found that only up to 41% of microglia expressed SALL1, TMEM119 and P2RY12. In addition, we could show that infiltrated macrophages also expressed these markers, clearly excluding cell specificity for microglia under tumor condition. In vitro, we detected all markers in microglia as well as macrophages. Analyses of a microglial cell line verified that glioma conditions led to reduced expression of SALL1, TMEM119 and P2RY12.

Consequently, the markers define the microglia population within the naïve brain and in the contralateral hemisphere but in the tumor area co-expression with microglia is inadequate. All analyzed markers cannot identify the whole entity of microglia intratumorally and peritumorally. In addition, infiltrated macrophages become positive for these markers in all brain regions. Our in vitro results complement the in vivo data. Here, both microglia and macrophages also show expression of the analyzed markers. Our results demonstrate that the investigated markers SALL1, TMEM119, P2RY12 and HEXB are not useful for differentiation of microglia and macrophages in the glioma context.

All markers showed mainly the expected cellular expression in epilepsy patient tissues and the naïve murine brains. Taking the technical limitations of immunofluorescence into account, SALL1 was found in the cell nucleus, TMEM119 and P2RY12 showed primarily membrane-bound expression, and HEXB appears to be expressed in the cytoplasm. However, for the macrophages in vitro, the BV2 cells and some IBA1^+^ cells in the tumor area there seems a shift in P2RY12 expression from the plasma membrane to the nuclei. P2RY12 is known as a receptor protein but it was described to be nuclear expressed in gliomas with high malignancy and this correlates with a more immunosuppressive phenotype of microglia^45^.

We observed IBA1-negative cells expressing the analyzed markers under both near-naïve and glioma conditions. In naïve brains and tissue from epilepsy patients, however, the identity of the additional stained cells beyond the IBA1-positive population remains unclear. Notably, epilepsy specimens exhibited more extensive positive staining than naïve murine brains. This may be explained by the fact that tissue from epilepsy patients cannot be considered entirely unaffected, as a certain degree of glial activation is known to occur in this condition^52^. RNA expression levels of the analyzed markers are generally low in brain cell types other than microglia^27,28,31,32,41^. However, our results suggest that under glioblastoma conditions some tumor cells may also express SALL1, TMEM119, and HEXB, and show high expression of P2RY12. Expression of these markers in tumor cells has been reported previously. For instance, SALL1 has been described as a tumor suppressor in several solid cancers^53^. TMEM119 expression has been detected in ovarian and gastric cancer cells^33,54^, while HEXB and P2RY12 have been reported to be expressed in human glioblastoma cells^55,56^. Therefore, partial staining of brain tumor cells with these markers may be expected.

SALL1 was first analyzed by Buttgereit et al.^27^. In the experimental setup of the study, the experimental autoimmune encephalomyelitis (EAE) model was used, representing a mouse model for multiple sclerosis, not for tumors^57^. Furthermore, to confirm microglia specificity bone marrow chimeras generated by TBI served as control. Among others, Müller et al. could show that using TBI leads to unspecific influx of macrophages due to impaired integrity of the blood brain barrier (Fig. 3d^17,50,58,59^, which may limit the significance of the results. In a second control, Buttgereit et al. referred to the CD45 expression levels to discriminate between CD45^low^ microglia and CD45^high^ macrophages. By now, several studies have proved the existence of CD45^high^ microglia under specific inflammatory conditions in the brain^16–20,60,61^. Therefore, this discrimination strategy is insufficient and underestimates the number of microglia in the tumor brain.

Caponegro et al. could show by immunofluorescence staining that TMEM119^+^ myeloid cells are present in the peritumoral and intratumoral area of murine glioblastoma tissues^62^. The authors used F4/80 for staining, and fewer F4/80⁺TMEM119⁺ cells were observed in the tumor core compared to the peritumoral area, which is consistent with our findings for IBA1 and TMEM119 co-staining. However, no additional analyses were performed to establish whether this marker is specific to microglia. In literature there is already evidence that macrophages are able to upregulate TMEM119^7^. Moreover, a study by Vankriekelsvenne et al.^63^ proved that TMEM119 is not exclusively expressed on microglia nor does it label all microglia cells. They used four different inflammatory animal models indicating a gradual loss of TMEM119-positivity following activation of microglia. A recently published review again points out the problems with TMEM119 as a single microglial marker under inflammatory circumstances^64^. In our study, we could show that validity of TMEM119 as a microglia specific marker in the tumor context is not given. This indicates that the glioma model leads to comparable activation status of all infiltrated myeloid cells.

P2RY12 was extensively described as a microglia marker by Haage et al.^31^. Although two tumor models were used and microglial expression was proven, identification of microglia and macrophages was partially done by CD45 expression level with the previously discussed issues leading to underestimation of resident microglia as CD45^high^ microglia were not considered^16–20,60–62^. Moreover, immunofluorescence staining indicated that P2RY12 expression is concentrated in the peritumoral region and largely absent within the tumor core^62^, in accordance with our data. Furthermore, detection of P2RY12 was done by gene expression analyses after cell sorting. Protein detection was therefore not provided in the glioma context. A review by Gómez Morillas clearly demonstrated that P2RY12 is highly expressed on non-activated quiescent microglia, but less expressed on activated microglia in a neuroinflammatory context, e.g. in CNS pathologies like multiple sclerosis or Alzheimer’s disease. In a tumor context, the P2RY12 expression is even negatively correlated with brain tumor malignancy of astrocytomas^41,65^. This also correlates with our results of decreasing expression towards the tumor core which obviously serves as an activation trigger. P2RY12 was already found on macrophages thereby acquiring features of tissue resident microglia^66,67^.

The marker HEXB is disqualified as a good microglial marker in our study by overall poor expression on naïve human and murine brain sections compared to good expression of the other microglial markers. We conducted different staining methods comparable to those done by other researchers^47^. Nevertheless, HEXB protein levels – which are expected to be upregulated in glioma specimen^47^– could not be effectively visualized in our study. Moreover, Zhu et al. demonstrated that HEXB is expressed in glioma, microglia and macrophages thereby indicating a lack of cell specificity^55^. Although the staining was technically feasible, the marker exhibited limited specificity and suboptimal sensitivity, as not all microglia-like cells were detected. The use of this marker cannot be recommended for microglial discrimination in either a naive context or in a tumor context.

At the same time, we must critically assess our differentiation strategy of bone marrow chimeras that served as control in this study^68^. In literature, either TBI or HPI models are used. These relatively complex methods are also prone to errors if TBI strategy was applied. This leads to unspecific macrophage influx, as we also observed for the contralateral hemisphere of TBI animals in contrast to the HPI model. This is most probably due to blood brain barrier alterations^17,50,58,59^ and it was proven that a significant amount of the CD11b^+^CD45^+^ myeloid cell population consists of macrophages lacking specific pathological stimulus^17^. But even if HPI irradiation strategy was chosen, immune cell activation was observed possibly influencing immune cell behavior^69^. Moreover, head protection includes also the neck region covering a second functional thymus in mice providing immune cells^70^. Consequently, reconstitution level could be impaired which limits the information value of our differentiation between microglia and macrophages. Nevertheless, microglia and macrophage distinction via HPI seem to be unambiguous compared to the classical CD45 expression level strategy as it would only underestimate the influx of peripheral macrophages to the diseased brain.

Our tested markers seem to lose microglia cell specificity upon glioma induced activation making a clear discrimination between microglia and macrophages difficult when using the markers. The gold standard of chimera generation using HPI should therefore still be considered, despite its considerable technical and temporal challenges. While several studies have demonstrated functional differences between glioma-associated microglia and macrophages^7,11,71^ accumulating evidence suggests that the phenotypic and functional states of tumor-infiltrating myeloid cells are primarily shaped by the tumor microenvironment rather than by ontogeny or origin^7,72^. Microglia, macrophages, and monocytes can exert overlapping immune-regulatory roles, adopting mixed or intermediate activation states beyond the traditional M1/M2 dichotomy, despite known differences in morphology and spatial distribution^67,73^. A study demonstrated that CCR2-deficiency impairs the migratory capacity of both microglia and macrophages without altering the total myeloid cell numbers or tumor characteristics, implying a compensatory equilibrium between these populations^19^. Similar findings were reported by Pombo Antunes et al.^26^ supporting the concept of functional redundancy within the glioma-associated myeloid compartment. Nevertheless, although these results emphasize that ontogeny alone does not dictate function, distinguishing microglia from macrophages remains relevant for therapeutic targeting. Lineage-specific vulnerabilities, such as signaling dependencies, metabolic pathways, or regional activation cues, may still provide valuable entry points for intervention. Hence, future therapeutic strategies should not focus solely on lineage or phenotype in isolation but rather integrate both perspectives to address the complex and plastic myeloid landscape in glioma. In summary, neither microglia specificity nor sensitivity of the examined markers — SALL1, TMEM119, P2RY12, and HEXB — is preserved under glioma or in vitro conditions, precluding their universal applicability for microglia identification.

## Supplementary Information

Below is the link to the electronic supplementary material.

Supplementary Material 1

## Data Availability

Data are available from the corresponding authors upon reasonable request.
