# Supplementary material for: Microglia-specificity of different markers is overridden in glioblastoma specimens
Source: Sci Rep. 2026 May 9;16:14687. doi: 10.1038/s41598-026-52315-y (PMC13157509; doi:10.1038/s41598-026-52315-y)
Supplement: Supplementary file 1 — Supplementary Material 1 [file 41598_2026_52315_MOESM1_ESM.docx]

**Figure S1**

**a**

**
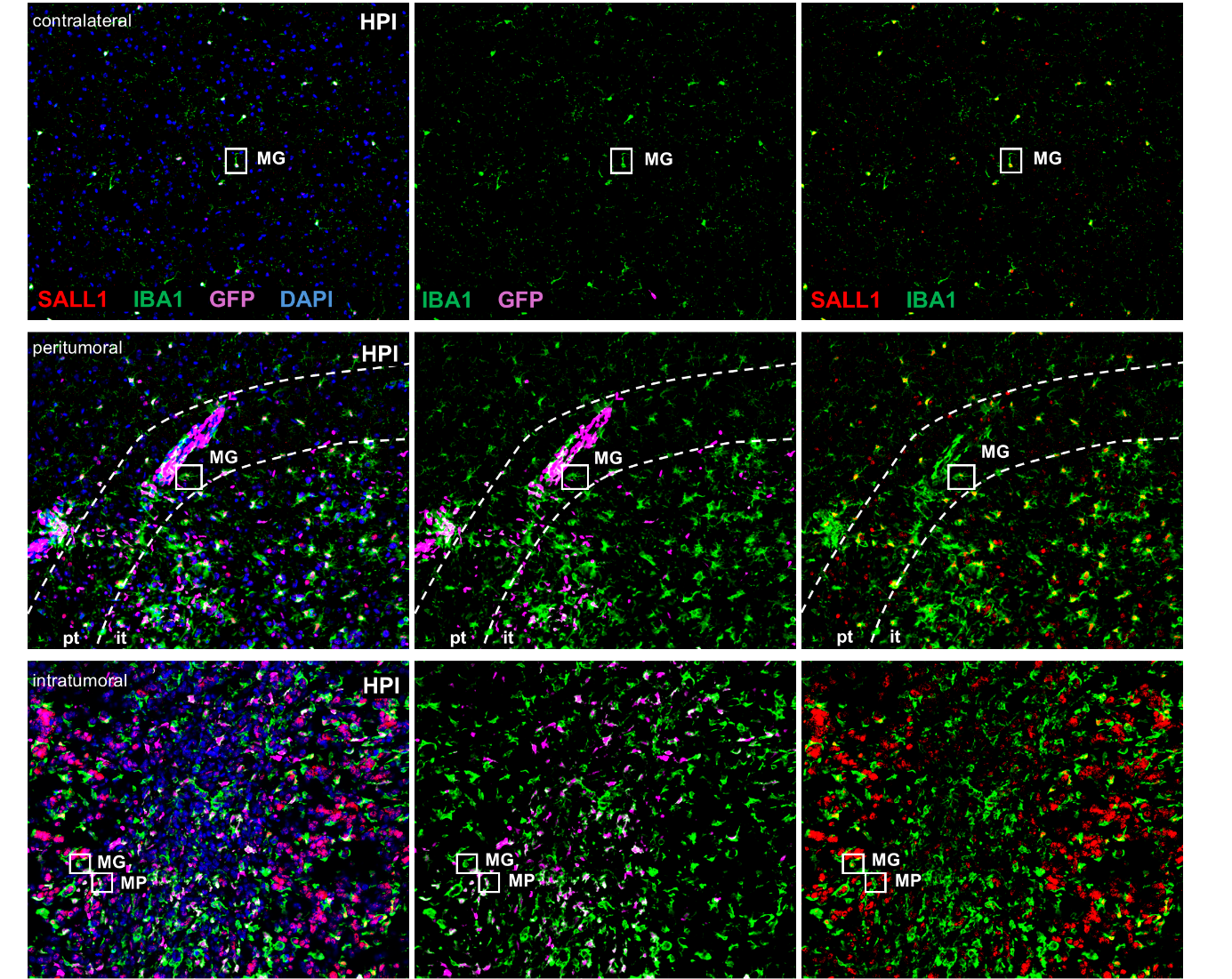
**

**b**

**
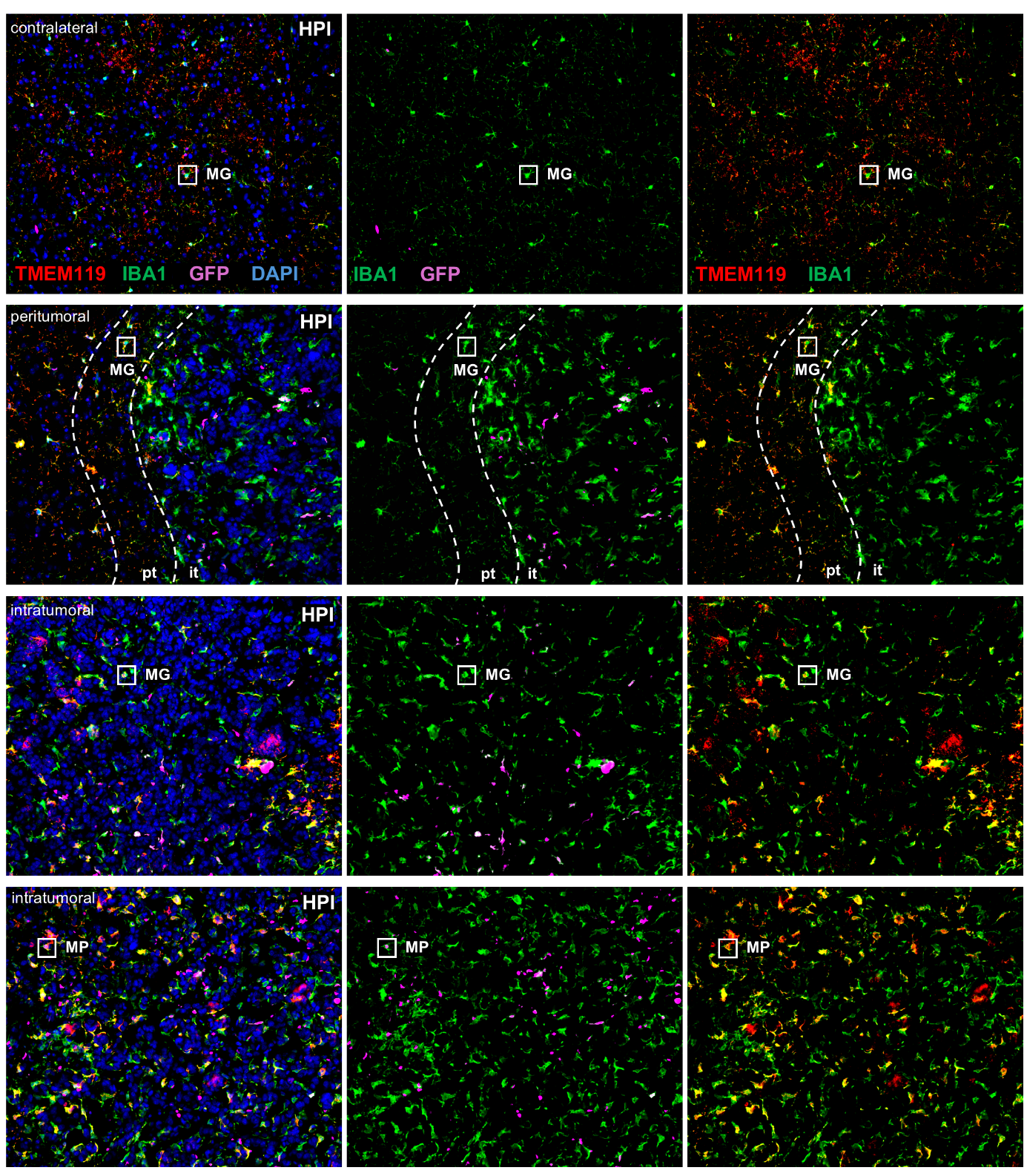
**

**c**

**
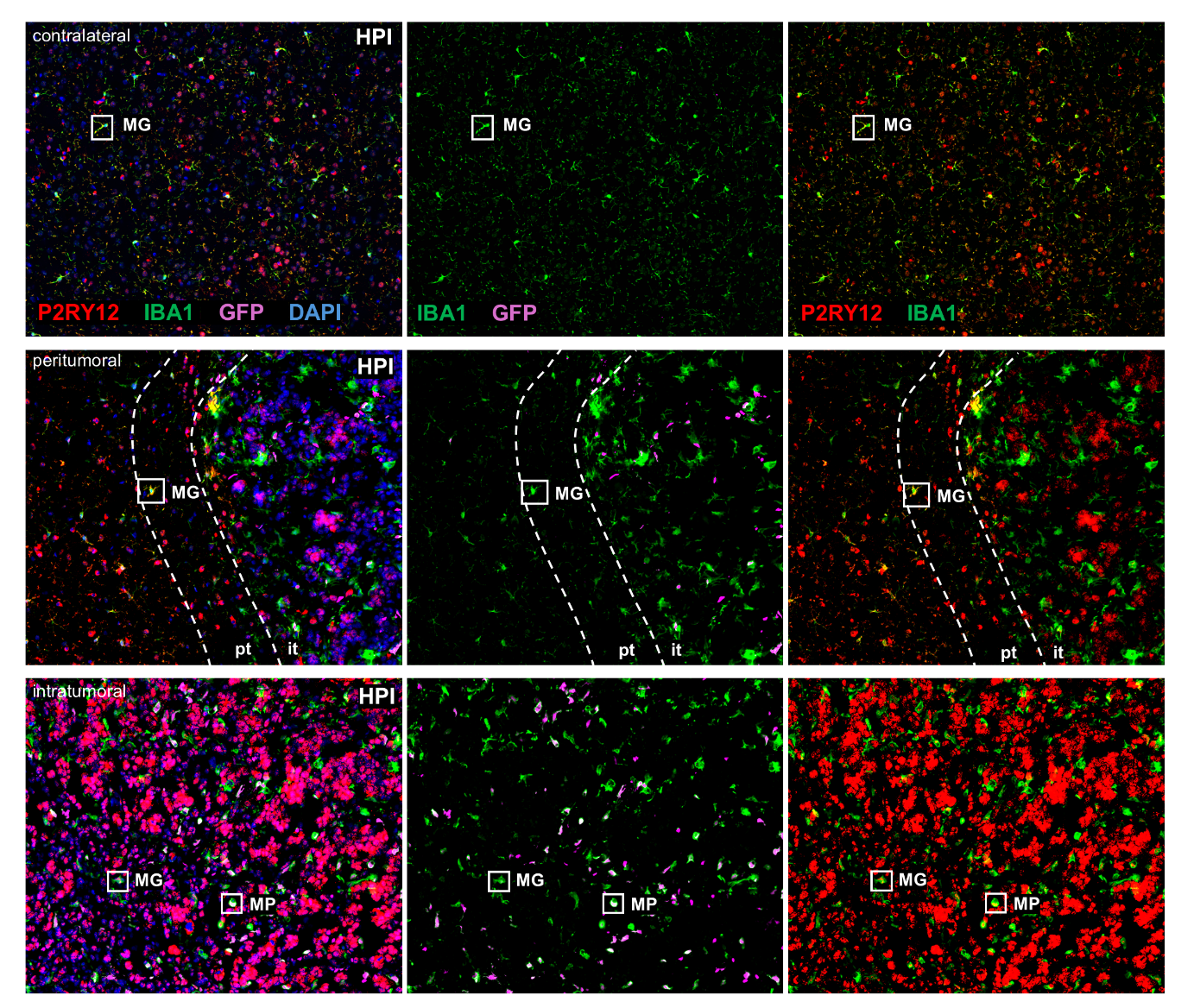
**

**Figure S1** Low-magnification images of stained brains of the head-protected irradiated mice. Brain sections of HPI chimeras were stained for IBA1 (green; microglia/macrophages), and the respective markers (red). **a** SALL1. **b** TMEM119. **c** P2RY12. Microglia (MG) and macrophages (MP) were discriminated by GFP expression (MG: IBA1^+^GFP^-^, MP: IBA1^+^GFP^+^). SALL1, TMEM119 and P2RY12 expression of IBA1^+^ cells in different brain regions under HPI condition. DAPI (blue, nuclei), GFP (violet, infiltrated cells). *HPI* head protected irradiation, *MG* microglia, *MP* macrophages, *pt* peritumoral (dashed line), *it* intratumoral, Squares indicate cells that are shown in main figure.

**Figure S2**

**a**

**
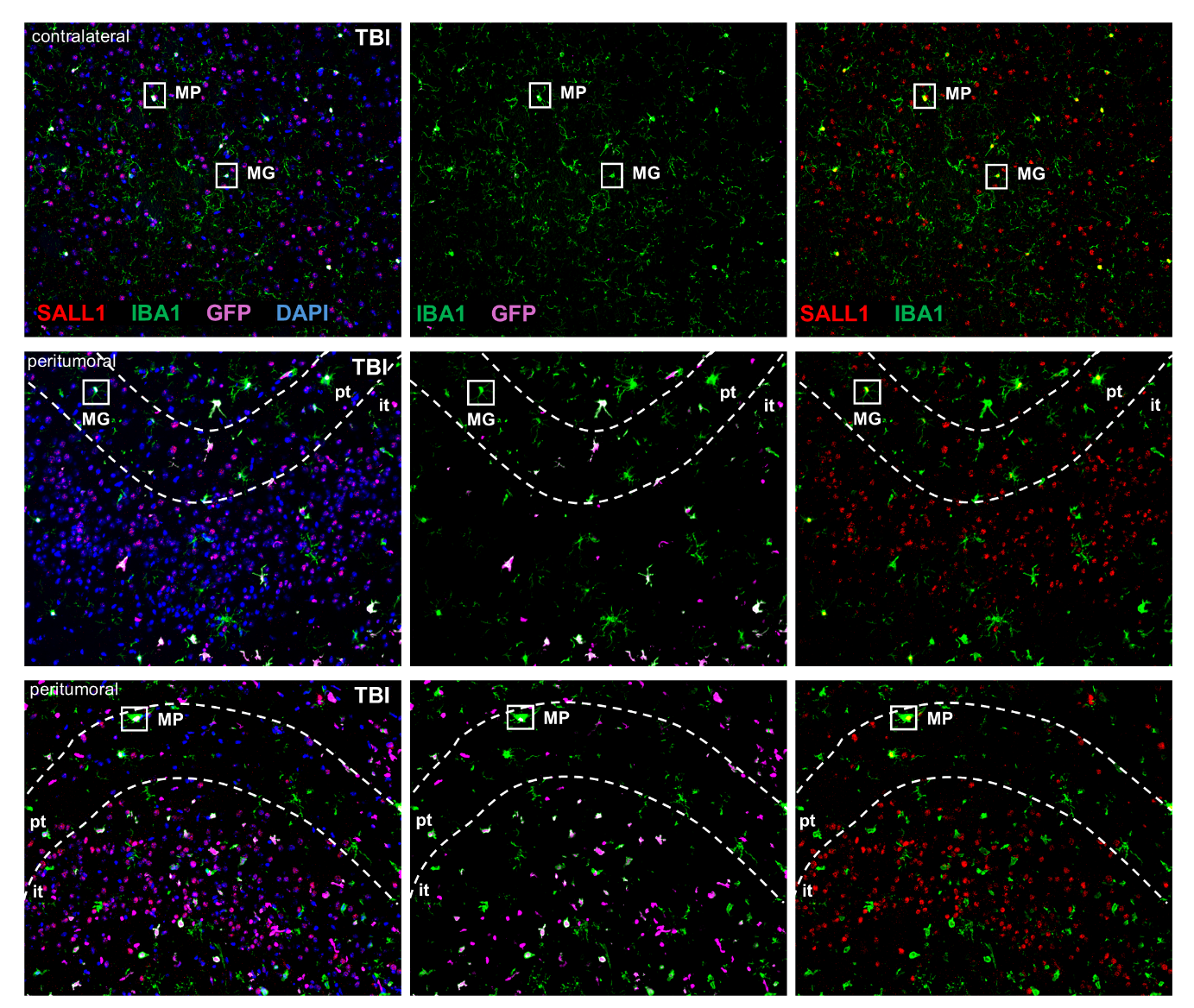
**

**a** (continued)


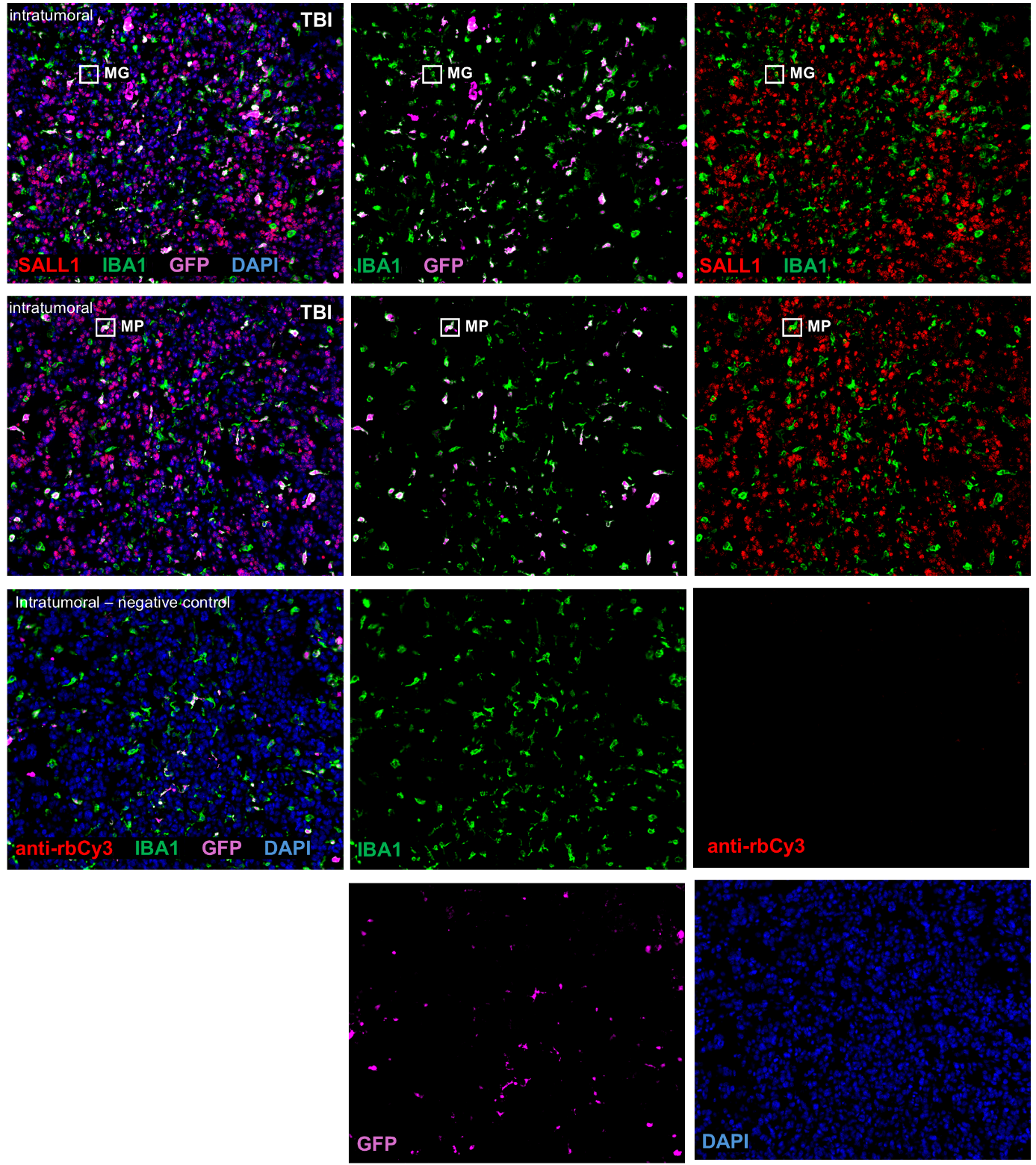


**b**

**
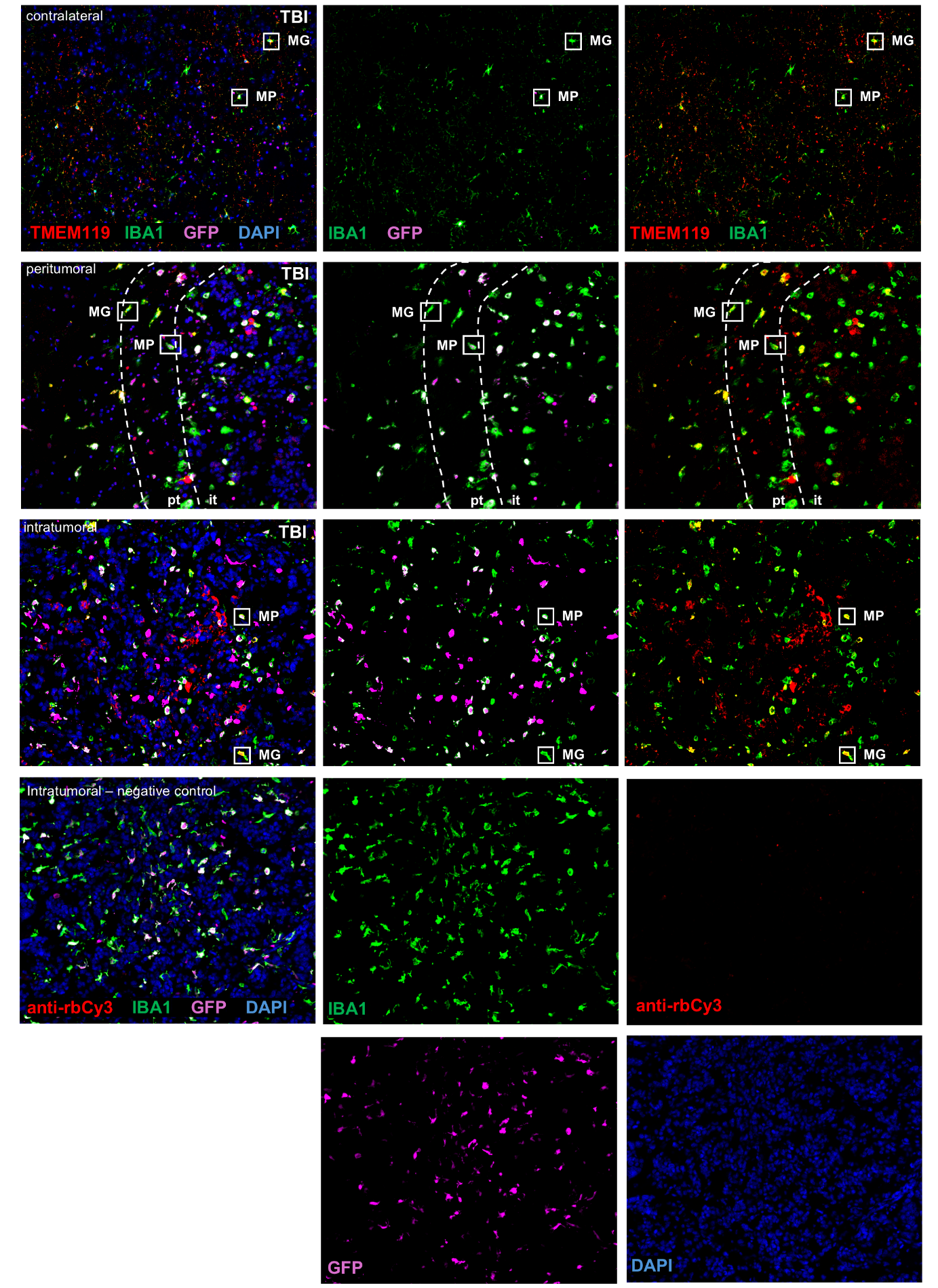
**

**c**

**
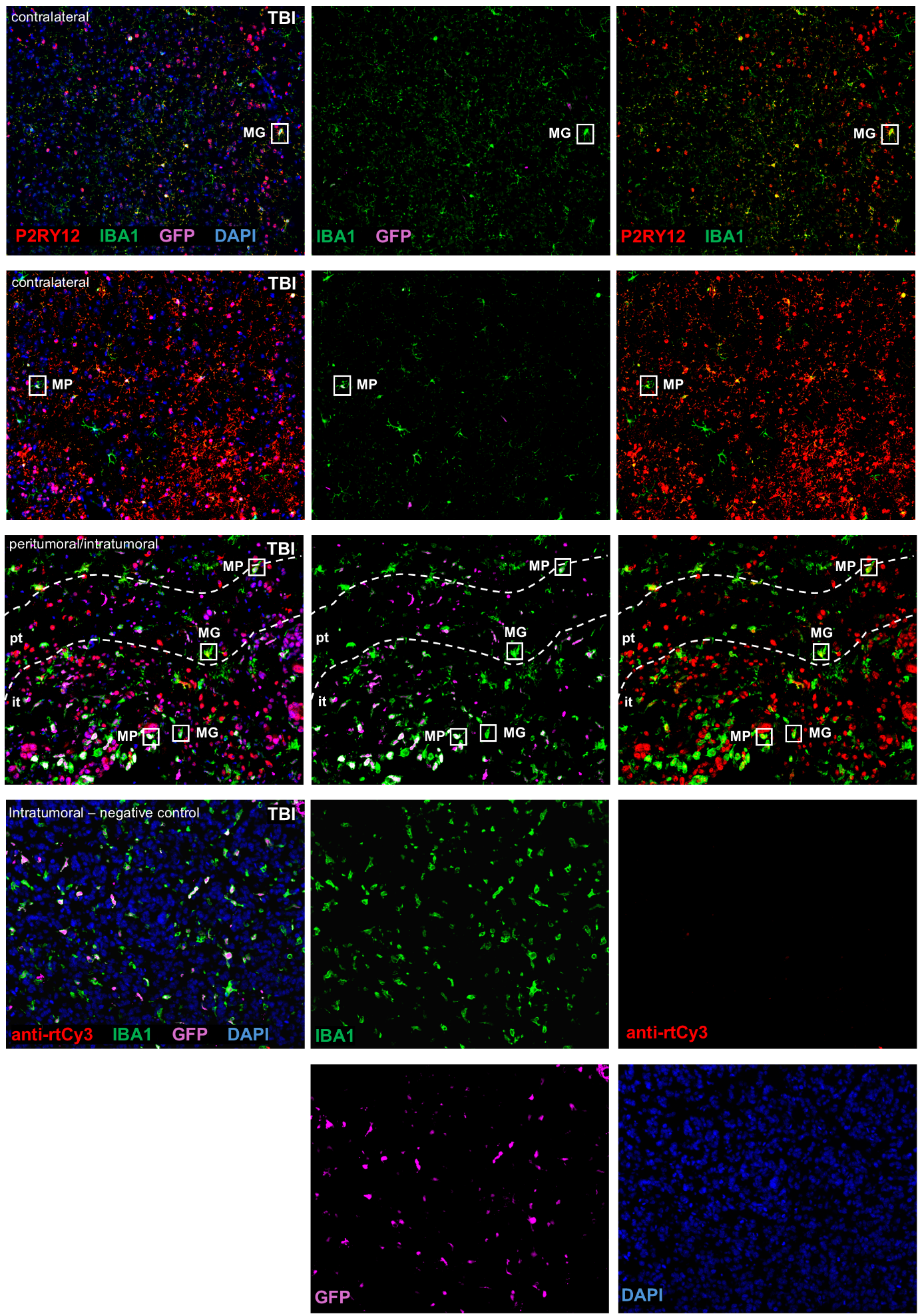
**

**Figure S2** Low-magnification images of stained brains of total body irradiated mice. Brain sections of TBI chimeras were stained for IBA1 (green; microglia/macrophages), and the respective markers (red). **a** SALL1. **b** TMEM119. **c** P2RY12. Microglia (MG) and macrophages (MP) were discriminated by GFP expression (MG: IBA1^+^GFP^-^, MP: IBA1^+^GFP^+^). SALL1, TMEM119 and P2RY12 expression of IBA1^+^ cells in different brain regions under TBI condition. Additionally, negative controls are presented (use of secondary antibodies without primary antibodies). Used secondary antibodies: anti-rabbit Cy3 (**a,b**) and anti-rat Cy3 (**c**). DAPI (blue, nuclei), GFP (violet, infiltrated cells). *TBI* total body irradiation, *MG* microglia, *MP* macrophages, *pt* peritumoral (dashed line), *it* intratumoral, *rb* rabbit, *rt* rat, *GFP* green fluorescent protein. Squares indicate cells that are shown in main figure.

**Figure S3**

**a**

**
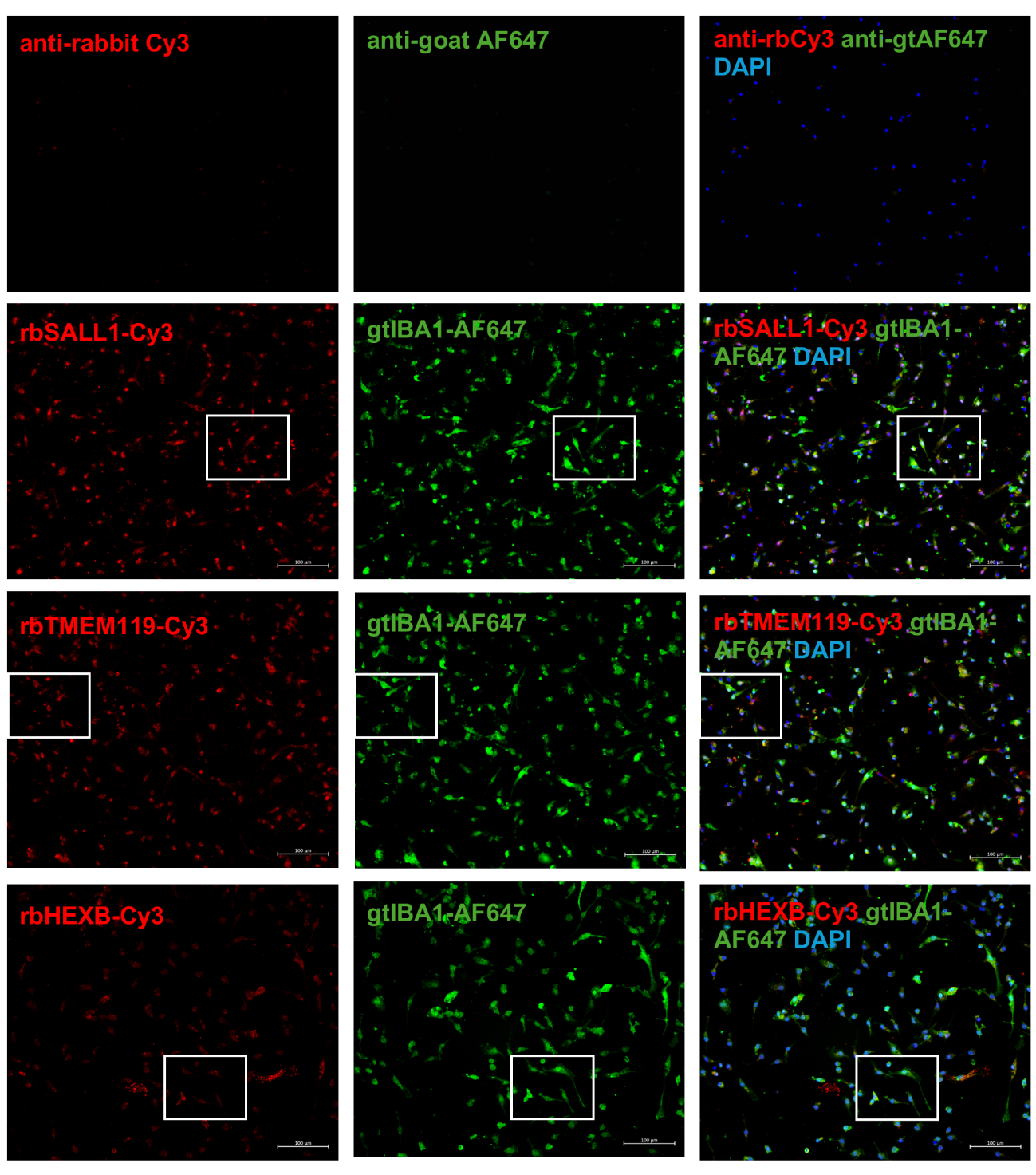
**

**b**

**
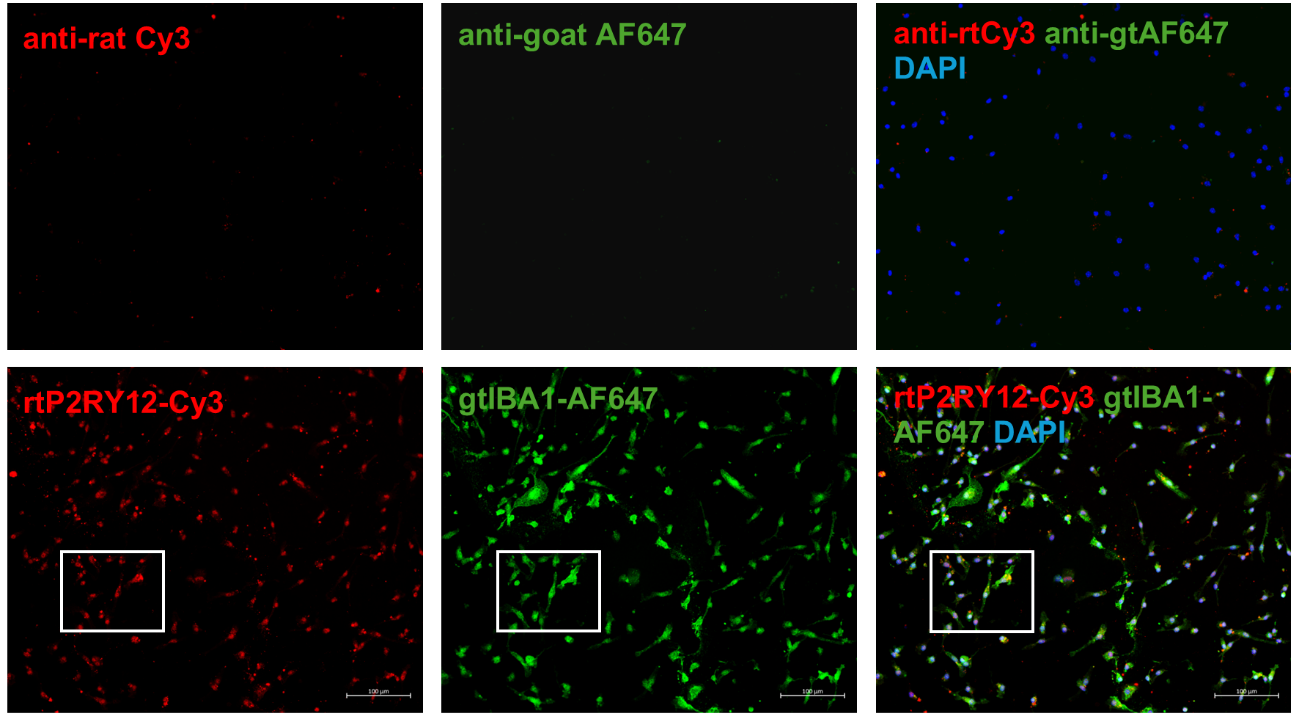
**

**c**

**
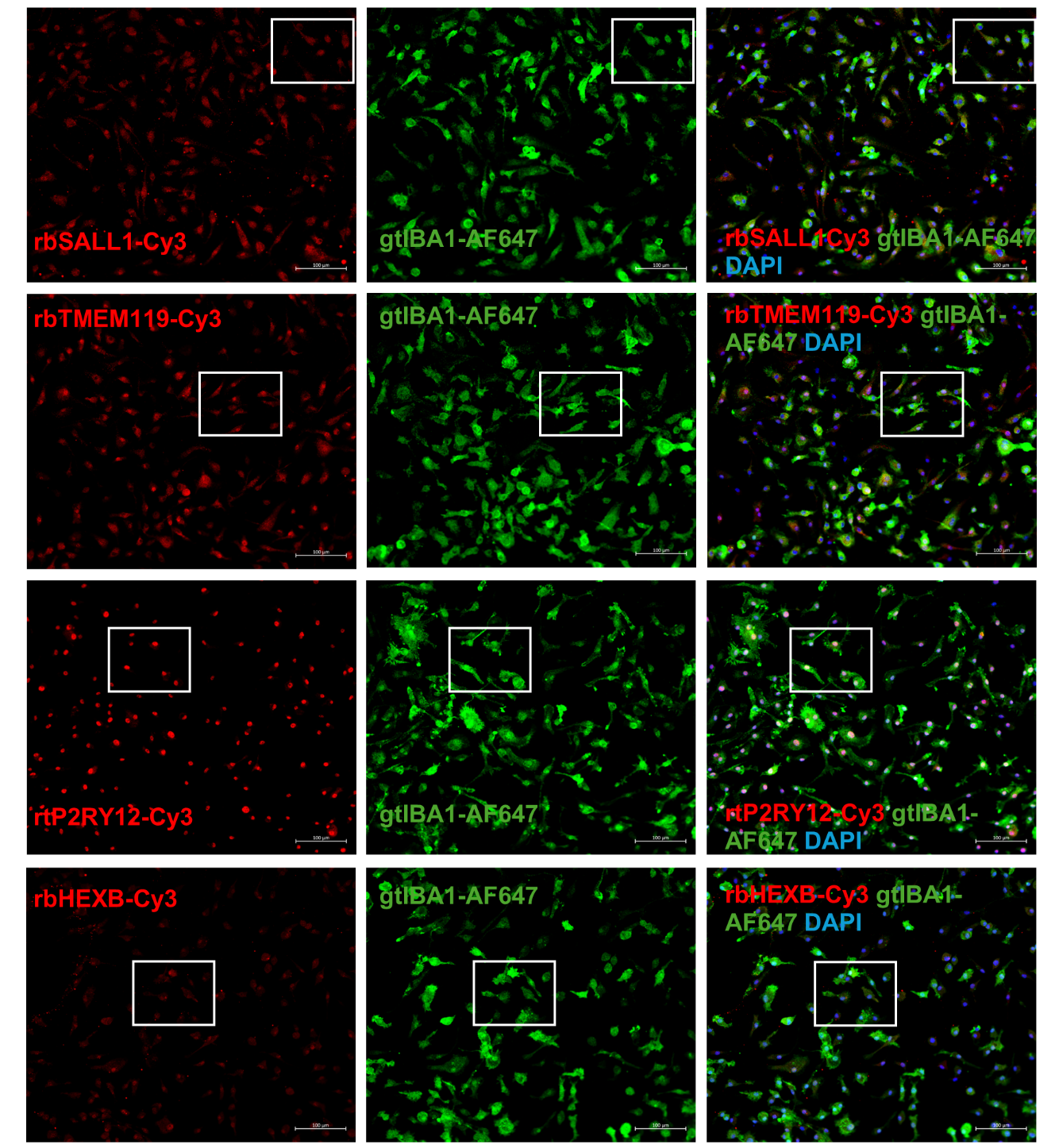
**

**Figure S3** Low-magnification images of primary microglia and primary macrophages expressing markers *in vitro*. **a,b** Microglia and **c** macrophages were stained for IBA1 (green; microglia/macrophages) and the respective markers (red). DAPI (blue; nuclei). Negative controls are included using only secondary antibodies anti-rabbit Cy3/anti-goat AF647 (**a,** first row) and anti-rat Cy3/anti-goat AF647 (**b,** first row). *Scale bars* 100µm. *MG* microglia, *MP* macrophages, *rb* rabbit, *rt* rat, *gt* goat. Representative pictures of two independent experiments are shown (3-4 wells/marker in total, 8 images/well). Rectangles indicate area that is shown in main figure.

**Figure S4**

**a**

**
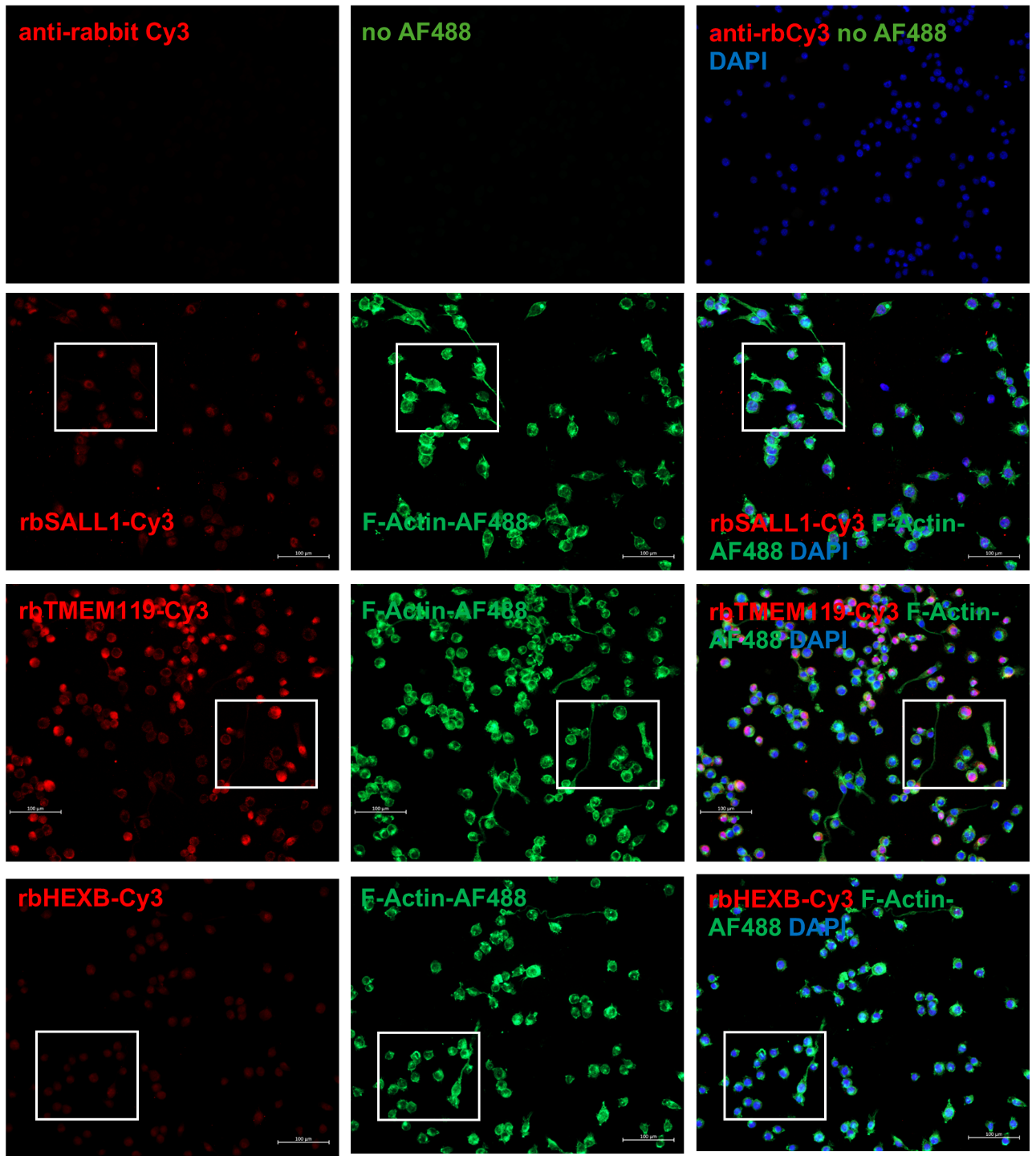
**

**b**

**
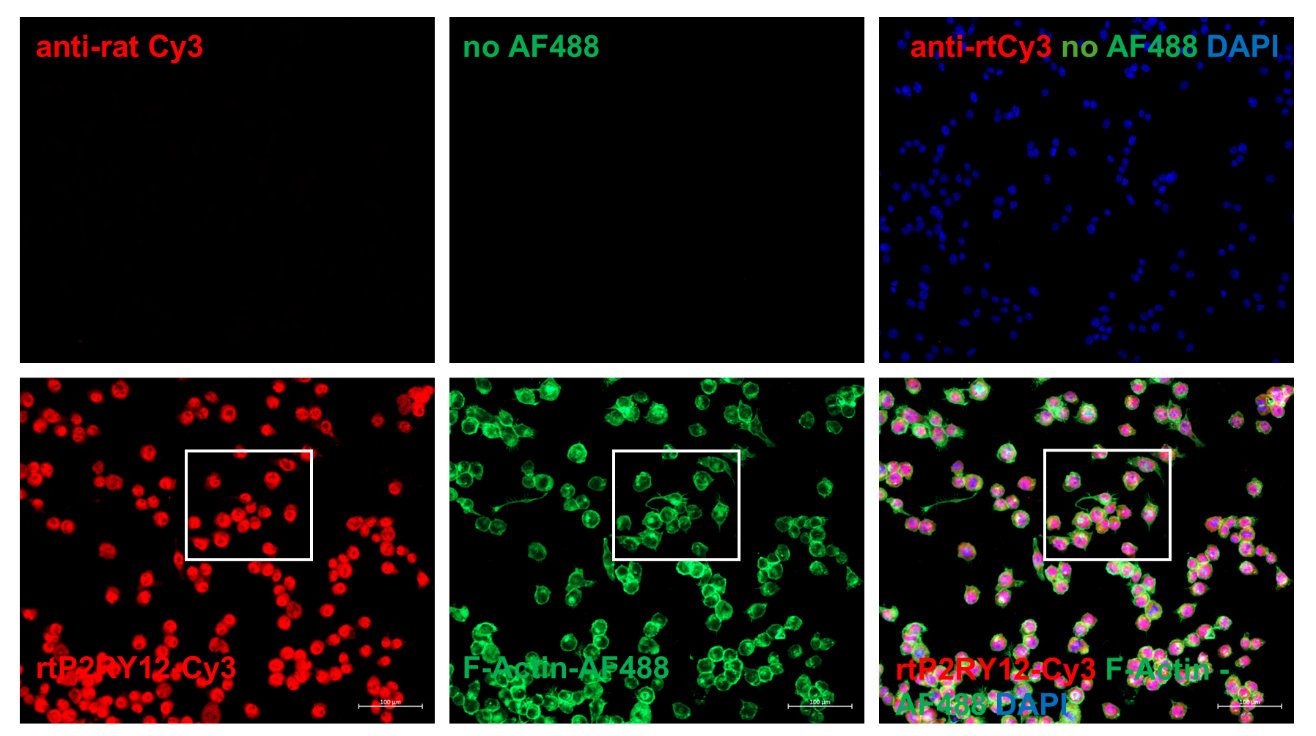
**

**c**

**
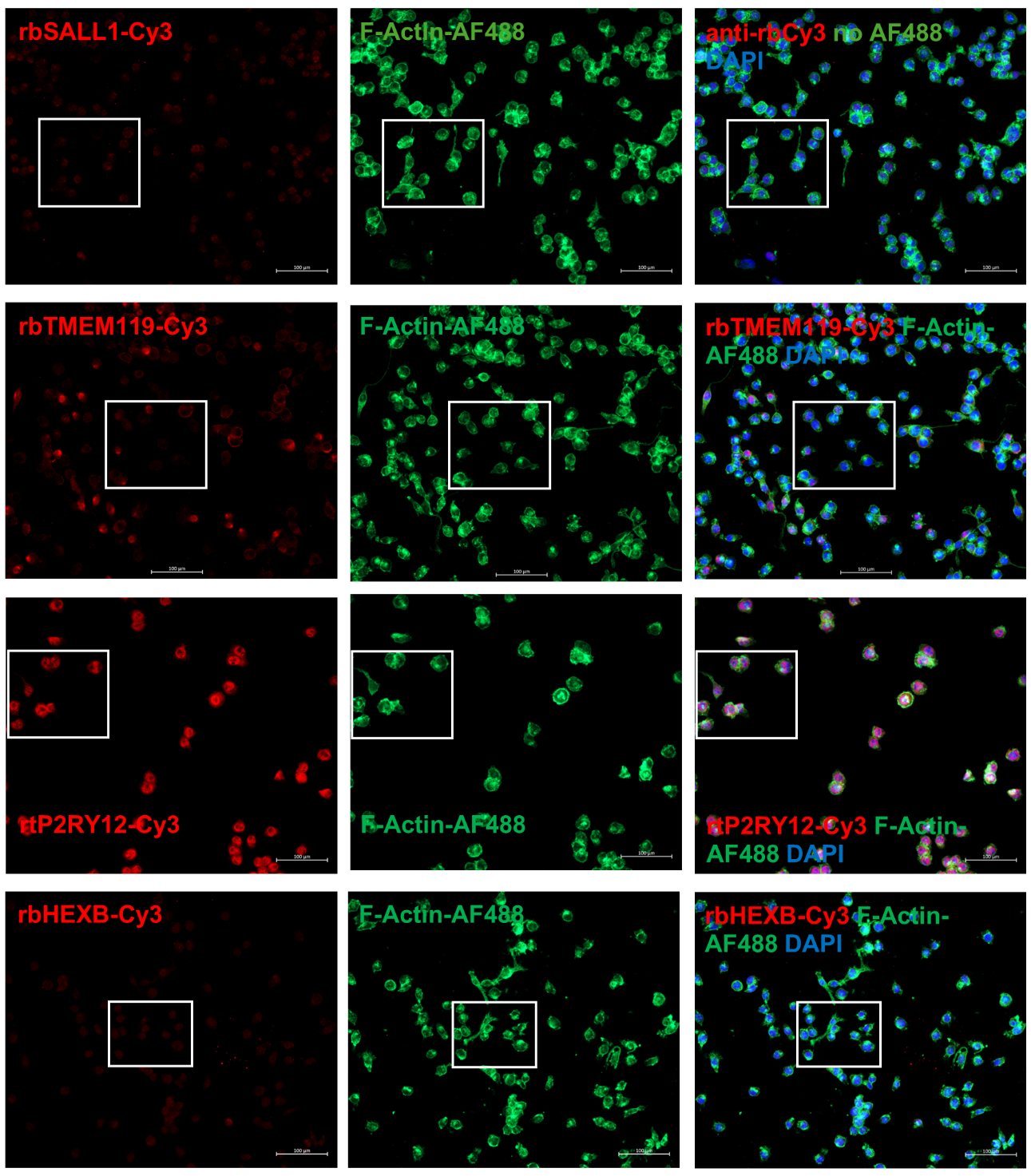
**

**Figure S4** Low-magnification images of microglia cell line (BV2) expressing markers *in vitro*. **a,b** BV2 cells were cultured for three days in standard medium (Control) or **c** tumor-conditioned medium. Cells were stained with Phalloidin (green, F-Actin) and the respective markers (red). DAPI (blue, nuclei). Negative controls are included using only secondary antibodies anti-rabbit Cy3/no Phalloidin (**a,** first row) and anti-rat Cy3/no Phalloidin (**b,** first row).  *Scale bars* 100µm. *rb* rabbit, *rt* rat. Representative images of three independent experiments are depicted. Rectangles indicate area that is shown in main figure.
